# Host receptor networking and molecular mechanisms of innate immune evasion in hepatitis E virus pathogenesis

**DOI:** 10.3389/fmicb.2026.1938537

**Published:** 2026-10-19

**Authors:** Ali Zohaib, Maham Yamin, Muhammad Usama Butt, Xing-Yi Ge, Safia S. Aljedani, Rwaa Hussin Abdulal

**Affiliations:** 1 Infectious Disease Research Department, King Abdullah International Medical Research Center, Jeddah, Saudi Arabia; 2 King Saud bin Abdulaziz University for Health Sciences, Jeddah, Saudi Arabia; 3 Ministry of the National Guard - Health Affairs, Jeddah, Saudi Arabia; 4 Department of Microbiology and Immunology, The Peter Doherty Institute for Infection and Immunity, The University of Melbourne, Melbourne, VIC, Australia; 5 State Key Laboratory of Virology and Biosafety and National Virus Resource Center, Wuhan Institute of Virology, Chinese Academy of Sciences, Wuhan, China; 6 Hunan Provincial Key Laboratory of Medical Virology, Hunan Research Center of the Basic Discipline for Cell Signaling, College of Biology, Hunan University, Changsha, China; 7 Infectious Disease Research Department, King Abdullah International Medical Research Center, Riyadh, Saudi Arabia; 8 King Saud bin Abdulaziz University for Health Sciences, Riyadh, Saudi Arabia; 9 Ministry of the National Guard - Health Affairs, Riyadh, Saudi Arabia

**Keywords:** cell entry, hepatitis E virus, *Hepeviridae*, host factors, immune evasion, innate immunity, interferon response, tissue tropism

## Abstract

Hepatitis E virus (HEV), the causative agent of hepatitis E, is a major global health concern, with seroprevalence estimates suggesting that approximately 939 million people worldwide have been exposed to the virus. Although historically regarded as a predominantly self-limiting cause of acute hepatitis, HEV is now recognized as a clinically diverse pathogen capable of causing acute liver failure, chronic hepatitis in immunocompromised individuals, and neurological, renal, hematological, and other extrahepatic manifestations. This review integrates current understanding of HEV cell entry, tissue and host tropism, innate immune recognition, and viral immune evasion. HEV exists in two particle forms: non-enveloped (nHEV) and quasi-enveloped (eHEV). Both use distinct but partially convergent entry pathways involving attachment factors, entry cofactors, endosomal trafficking machinery, and lysosomal processing rather than a single canonical receptor. We evaluate the evidence supporting established and candidate host factors and propose a dual-module heuristic to organize current receptor evidence while emphasizing that this framework remains provisional and requires validation in physiologically relevant models. We further examine how viral genetic determinants, receptor engagement, post-entry replication compatibility, and host antiviral responses collectively shape tissue tropism and cross-species transmission. Innate recognition involves RIG-I and MDA5 signaling through MAVS–TBK1–IRF3, alongside TLR pathways converging on IRF3 and NF-κB, driving type I and III interferon responses. HEV, however, counteracts these defenses at multiple levels. Functional regions within ORF1 interfere with pathogen sensing and interferon signaling; ORF2 contributes to immune evasion through a secreted glycosylated decoy isoform and modulation of NF-κB signaling; and ORF3 exerts context-dependent immunomodulatory effects while facilitating quasi-enveloped virion egress through the ESCRT pathway. Together, these mechanisms support a model in which HEV induces a sustained but incompletely effective antiviral response that may permit viral persistence, particularly under immunosuppression. Integrating entry biology with tropism, zoonotic determinants, and innate immune evasion highlights key unresolved mechanisms and provides a framework for developing improved experimental models, antiviral strategies, and diagnostic approaches for hepatitis E.

## 1 Introduction

Hepatitis E virus (HEV) was first identified in the 1980s following an unexplained waterborne hepatitis outbreak and has since been recognized as a leading cause of acute viral hepatitis worldwide (Webb and Dalton, 2019; Raji et al., 2022). The global prevalence of HEV-associated hepatitis varies significantly between developing and developed countries. This is primarily influenced by the distinct modes of viral transmission and the presence of reservoir hosts (Pérez-Gracia et al., 2015; Aslan and Balaban, 2020; Özcan et al., 2024). Enteric transmission through contaminated water sources is the primary mode of disease spread in endemic regions. However, zoonotic transmission from domestic and wild animals also contributes significantly to sporadic cases (Blasco-Perrin et al., 2016; Letafati et al., 2024; Tene et al., 2025). The global burden of hepatitis E exhibits two distinct patterns, which are largely defined by sanitary conditions and socioeconomic status (Hughes et al., 2010; Raji et al., 2022; Letafati et al., 2024). In developing countries, large waterborne outbreaks of hepatitis E are well documented, and the virus additionally accounts for more than 50% of acute viral hepatitis cases in endemic regions, with notably high morbidity and mortality in pregnant women (Pérez-Gracia et al., 2017; Zahmanova et al., 2023). On the other hand, sporadic cases linked to zoonotic transmission have been reported in developed countries. However, due to poor awareness and a lack of routine diagnostic screening, the global burden of hepatitis E is significantly underestimated.

A 2020 systematic review and meta-analysis concluded that approximately 939 million people (one in every eight individuals globally) have been previously exposed to HEV based on seroprevalence data. This study further states that, an estimated 15–110 million have a recent or ongoing infection (Li P. et al., 2020).

The most recent Global Burden of Disease analyses indicate that HEV incidence has only marginally declined between 1990 and 2021, from 269.68 to 260.41 per 100,000. However, it is projected to rebound to approximately 267 per 100,000 by 2030 (Wu et al., 2025).

Despite its global significance, the molecular mechanisms underlying HEV’s ability to establish infection, evade host immunity, and persist in vulnerable populations remain incompletely understood. Moreover, no recent synthesis has connected viral entry, tissue tropism and zoonotic determinants, and the layered innate immune-evasion program within a single mechanistic account, as recent reviews have addressed entry factors and assay systems (Brüggemann et al., 2025), virion-form-specific entry (Shahini et al., 2024), innate immune sensing (Devhare et al., 2021), and the zoonotic landscape (Alkan et al., 2025) largely in isolation. The remainder of this review therefore transitions from the epidemiological burden outlined above to the molecular life cycle, proceeding in a deliberately cumulative sequence in which each section provides the conceptual groundwork for the next. We first consolidate the virological platform, clinical spectrum, genetic classification, virion architecture, and genome organization, since the biophysical form of the particle naked (nHEV) versus quasi-enveloped (eHEV) and the three open reading frames it encodes ultimately dictate both entry strategy and immune antagonism (Nagashima et al., 2017; Kenney and Meng, 2019). The review then develops the receptor-network paradigm: in contrast to hepatitis C virus, for which essential entry receptors such as CD81 and claudin-1 are clearly defined (Gerold et al., 2020), no single canonical HEV receptor has been identified.

Instead, nHEV and eHEV virions engage a hierarchical portfolio of attachment factors, entry cofactors, endocytic routes, and lysosomal processing machinery (Brüggemann et al., 2025), evidence that we organize into a dual-module heuristic comprising a ubiquitous core module mediating basal entry across nucleated cells and a liver-enriched ASGPR–EGFR module that may act as a tissue-specific amplifier of entry a designation that is deliberately qualified, since EGFR functions in a stage-dependent manner across the HEV life cycle: although EGFR engagement facilitates viral entry into human hepatocytes (Schrader et al., 2023), HEV-induced EGFR downregulation favors viral replication, release, and suppression of innate immunity (Woytinek et al., 2024), such that EGFR inhibition can impair binding and entry yet still increase extracellular viral titers (Schrader et al., 2023; Woytinek et al., 2024).

This receptor-level framework then serves as the interpretive lens for the analysis of tissue tropism and zoonotic spillover why HEV productively infects a broad range of cells and hosts despite lacking a hepatocyte-exclusive receptor (Nan and Zhang, 2016; Yadav and Kenney, 2023; Brüggemann et al., 2025), and how post-entry replication compatibility and cell-intrinsic antiviral responses refine species barriers. Having established how HEV enters cells and spreads across tissues, the review shifts to host defenses, characterizing innate immune sensing through RIG-I, MDA5, and TLR pathways and the type I/III IFN–ISG response these sensors mobilize (Sooryanarain et al., 2020; Xu et al., 2021), before deconstructing the layered evasion program in which ORF1, ORF2, and ORF3 disable distinct nodes of the antiviral cascade from PRR engagement and the MAVS–TBK1–IRF3 signalosome (Nan et al., 2014b; Kang et al., 2018), to the antigen-decoy function of the secreted ORF2S isoform (Yin et al., 2018; Walker, 2019), to ORF3-mediated modulation of innate immune signaling, including TLR7-mediated suppression of type I IFN, coupled with quasi-enveloped egress (Nagashima et al., 2011; Lei et al., 2018).

Cumulatively, these sections trace a single mechanistic arc from cell entry, to tissue and cross-species spread, to innate immune escape that frames the unresolved questions and experimental strategies taken up in the closing sections. As a working synthesis of findings derived from separate experimental systems, the dual-module heuristic is intended to guide future hypothesis testing rather than to function as an established causal mechanism and requires validation in physiologically relevant models. While adaptive immunity undeniably shapes infection outcomes, this review focuses deliberately on the innate evasion mechanisms that establish the primary viral foothold.

### 1.1 Scope and literature selection

This review focuses on host receptor engagement and innate immune evasion in HEV pathogenesis. Literature was identified through searches of PubMed, Google Scholar, and Scopus using combinations of the terms hepatitis E virus, ORF1/ORF2/ORF3, entry receptor, heparan sulfate proteoglycan, ASGPR, integrin, NPC1, innate immunity, interferon, immune evasion, extrahepatic tropism, and zoonosis, together with forward and backward citation tracking of the retrieved articles. We prioritized primary experimental studies and the current ICTV taxonomy. Studies were included if they reported original experimental, clinical, epidemiological, or taxonomic data on HEV. Articles published in languages other than English were generally excluded, with the exception of one relevant study evaluated via its English abstract; where critically relevant, selected non-peer-reviewed preprints were also considered.

## 2 Clinical spectrum of HEV infection

Most HEV infections are asymptomatic and resolve spontaneously. However, HEV infection can cause acute and chronic hepatic disease, along with extrahepatic manifestations. Infection with genotypes 1 and 2 can lead to acute liver failure with high case fatality rates in high-risk populations (Alexandrova et al., 2024). HEV-associated chronic hepatitis is well documented in immunocompromised individuals, especially in solid organ transplant recipients, patients with hematologic malignancies, and persons with advanced HIV infection. The disease can progress to cirrhosis and liver failure (Kamar et al., 2013a,b; Lhomme et al., 2020). Genotypes 3 and 4 have been identified in the majority of chronic cases (Horvatits et al., 2019; Lhomme et al., 2020). HEV infection during pregnancy is associated with a significantly elevated risk of acute liver failure (ALF), preterm delivery, stillbirth, and neonatal hepatitis. Genotype 1 has been predominantly identified in pregnancy-related hepatitis E in endemic regions. Vertical transmission of the virus has also been reported (Khuroo et al., 2009; Aslan and Balaban, 2020; Khuroo, 2021).

Over the past decade, HEV infection has been increasingly linked to neurological syndromes such as Guillain–Barré syndrome, neuralgic amyotrophy, and encephalitis. HEV infection has also been linked to renal injury, pancreatitis, and a range of hematological disorders, including thrombocytopenia and cryoglobulinemia (Kamar et al., 2016; Fousekis et al., 2019; Velavan et al., 2021). Taken together, this expanding clinical picture has fundamentally repositioned HEV from a self-limiting hepatic illness to a systemic infection with consequences well beyond the liver.

## 3 Genetic diversity and classification of HEV

Understanding the diversity of HEV requires moving through several levels of classification, from the family level down through genus, species, and finally the individual genotypes most relevant to human disease. HEV is a single-stranded, positive-sense RNA virus of the *Hepeviridae* family. The *Hepeviridae* family contains two subfamilies (*Orthohepevirinae* and *Parahepevirinae*). *Orthohepevirinae* is further subdivided into four genera (*Paslahepevirus, Rocahepevirus, Chirohepevirus* and *Avihepevirus*). Of these four, *Paslahepevirus* and *Rocahepevirus* are the most clinically relevant (Figure 1). Both contain lineages capable of infecting humans (Smith et al., 2020; Purdy et al., 2022; Benavent et al., 2023; Rivero-Juarez et al., 2026). *Paslahepevirus balayani* is the most clinically relevant species and can infect humans and a wide range of domestic and wild animals (Table 1). Based on whole-genome sequence analysis, members of this species have been assigned to eight different genotypes, HEV-1 to HEV-8. Of these eight genotypes, HEV-1 and HEV-2 are restricted to humans (Letafati et al., 2024). Genotype 1 is also distinguished at the molecular level by a fourth, genotype-specific open reading frame (ORF4), which enhances viral replication under conditions of endoplasmic reticulum stress and is absent from genotypes 3 and 4 (Nair et al., 2016). HEV-3 and HEV-4 are zoonotic genotypes and circulate among domestic pigs and wild boars. Human infection with HEV-1 and HEV-2 primarily occurs through the consumption of contaminated water, whereas for genotypes 3 and 4, major sources of infection include undercooked pork products or direct contact with infected animals (Letafati et al., 2024). Genotypes 1 and 2 are endemic in developing regions of Asia, Africa, and Mexico (Lu et al., 2006; Letafati et al., 2024). HEV-3 has a worldwide distribution, whereas HEV-4 is predominantly found in Asia (Lu et al., 2006; Anheyer-Behmenburg et al., 2017; Figure 2). Studies have identified genotypes 5 and 6 only in wild boar and genotypes 7 and 8 in dromedary and Bactrian camels, respectively. Of these, HEV-7 has been associated with chronic human infection in immunocompromised patients following the consumption of camel milk and meat (Anheyer-Behmenburg et al., 2017; Sridhar et al., 2017; Santos-Silva et al., 2023; Alkan et al., 2025).

**FIGURE 1:**
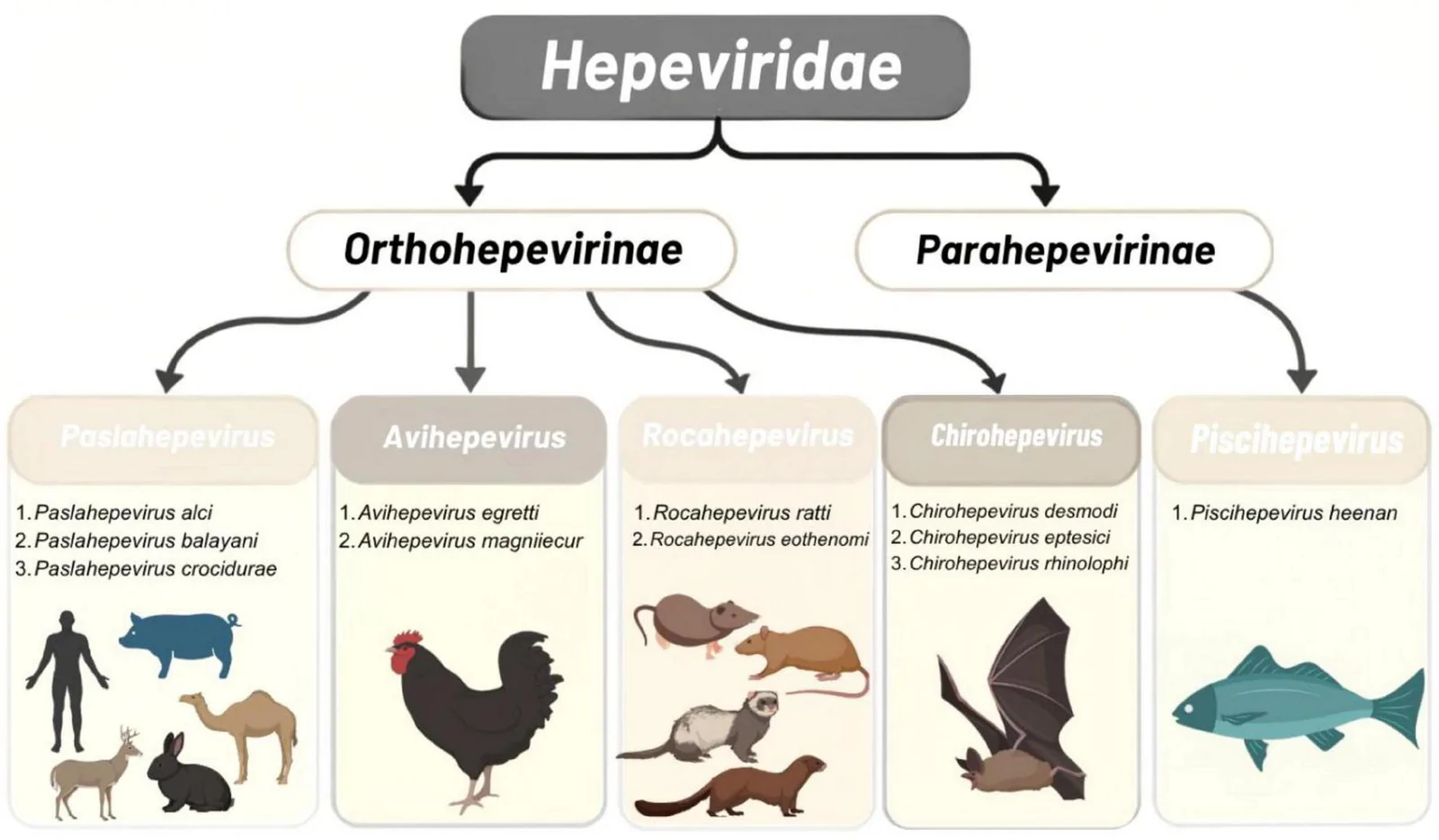
Taxonomic classification of the family *Hepeviridae*. Animal illustrations were created using BioRender.

**TABLE 1:** Clinically and epidemiologically relevant HEV genotypes, their known hosts/reservoirs, geographic distribution, and documented human infections.

HEV genotype/ lineage	Host/known reservoir	Geographic distribution	Documented human infections
HEV-1	Humans	Asia, Africa (endemic regions)	Yes: acute hepatitis; waterborne outbreaks and epidemics.
HEV-2	Humans	Africa, Mexico	Yes: acute hepatitis; waterborne outbreaks.
HEV-3	Humans, pigs, wild boar, deer, rabbits, mongooses	Worldwide	Yes: zoonotic, foodborne; acute hepatitis, chronic infection in immunocompromised patients.
HEV-4	Humans, deer, pigs, wild boar, etc.,	Asia, Europe	Yes: zoonotic; acute hepatitis, chronic infection in immunocompromised patients.
HEV-5	Wild boar	Japan	No: animal-associated; zoonotic potential uncertain.
HEV-6	Wild boar	Japan	No: animal-associated; zoonotic potential uncertain.
HEV-7	Dromedary camel	Middle East, North Africa, Pakistan	Yes: one documented human infection in a liver-transplant recipient; zoonotic transmission suspected following consumption of camel meat.
HEV-8	Bactrian camel	China	No: no documented human infections; experimental infection of cynomolgus macaques resulted in acute and chronic hepatitis, suggesting potential for zoonotic transmission.
HEV-C1	Rats and shrews	Widespread across Asia, Europe, Americas, Africa	Yes: more than 50 confirmed human cases, including acute, severe/fatal, and chronic hepatitis in immunocompetent and immunocompromised patients.
HEV-C2	Ferrets, mink	Netherlands, USA, Japan, Denmark, China	No: no human infections reported; negligible zoonotic potential.
HEV-C3 to HEV-C5	Rodents	China; scattered detections elsewhere	No: no human infections reported; negligible zoonotic potential.

HEV-1 to HEV-8 correspond to genotypes within *Paslahepevirus balayani*, while HEV-C1 to HEV-C5 represent lineages associated with *Rocahepevirus ratti.*

**FIGURE 2:**
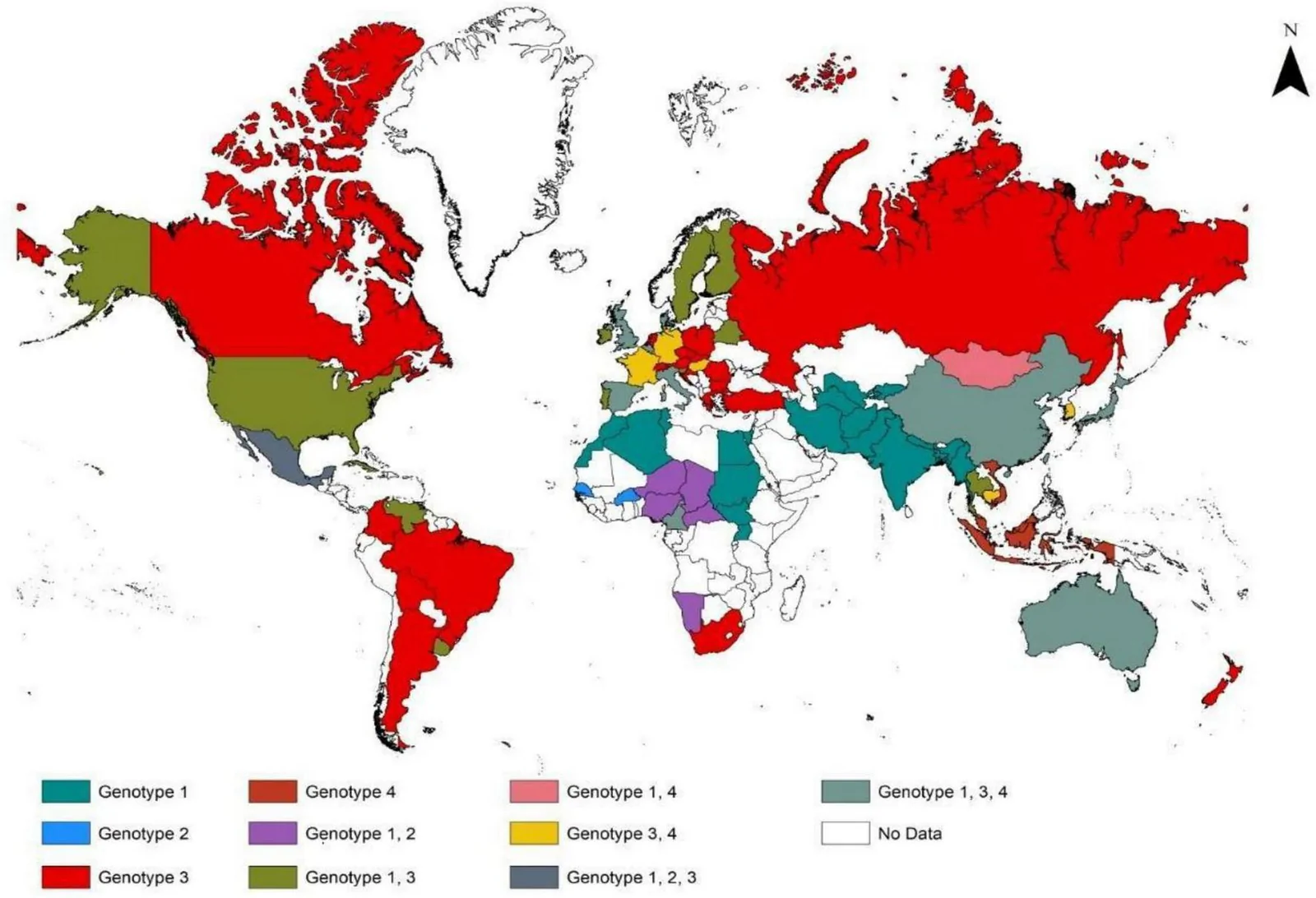
Global geographic distribution of hepatitis E virus (HEV) genotypes 1–4 based on confirmed human genotype data. The map excludes environmental and animal surveillance data. No Data indicates regions without available confirmed human genotype data.

All eight genotypes of HEV within *Paslahepevirus balayani* belong to a single serotype, and researchers have subdivided them into subtypes (Lorusso et al., 2024). The modern HEV subtyping system is the result of iterative consensus work coordinated by the ICTV *Hepeviridae* Study Group. The original subtyping classification system recognizes 24 subtypes across HEV-1–HEV-4 (Lu et al., 2006). Later, in 2020, Smith and colleagues updated the 2016 table of HEV subtype reference sequences. They classified HEV into eight genotypes and 36 subtypes, though several genomes remain unassigned pending broader sequence data (Smith et al., 2016, 2020; Letafati et al., 2024).

The genus *Rocahepevirus* contrasts sharply with *Paslahepevirus* at the molecular level, a distinction that is central to appreciating HEV as a systemic pathogen. Its representative species, *Rocahepevirus ratti*, commonly referred to as rat HEV, is genetically highly divergent from the *Paslahepevirus* genotypes that cause classical human hepatitis E (Yadav et al., 2025). Rat HEV is categorized into five genotypes labeled C1 through C5 (Rivero-Juarez et al., 2026). While genotype C1 infects rats and shrews and C2 is limited to mustelids such as ferrets and minks (De Sabato et al., 2020; Rivero-Juarez et al., 2026), three other rodent-related strains have been discovered through wildlife and environmental monitoring. Previously, members of *Rocahepevirus* were believed to be non-pathogenic to humans. More recently, however, the zoonotic potential of rat HEV has been recognized, especially for genotype C1. Genotype C1 can cross species barriers and is pathogenic to humans, with documented infections in both immunocompetent and immunocompromised individuals (Yadav et al., 2025). First identified in 2010 in wild rats in Germany, rat HEV was initially regarded as a rodent-restricted virus with no relevance to human health; this assumption collapsed in 2018, when the first confirmed human infection was reported in Hong Kong in a liver transplant recipient with chronic hepatitis of unknown origin (Rivero-Juarez et al., 2026). Subsequent case reports, including one from Canada, demonstrated that zoonotic spillover is not confined to immunocompromised hosts, and retrospective analyses and active surveillance have since identified more than 50 confirmed human cases across Asia and Europe, establishing rat HEV as a zoonotic virus with sustained human transmission rather than a sporadic spillover event (Rivero-Juarez et al., 2026). *Rocahepevirus ratti* has accordingly emerged as a distinct cause of acute hepatitis worldwide (Benavent et al., 2023), with clinical outcomes ranging from acute–occasionally severe or fatal hepatitis in immunocompetent individuals to persistent chronic hepatitis in immunosuppressed patients, predominantly transplant recipients and people living with HIV (Sridhar et al., 2018; Rivero-Juarez et al., 2026). This poses an emerging threat, as zoonotic rat HEV can bypass routine diagnostics designed for conventional HEV detection.

The zoonotic capacity of rat HEV is mirrored and mechanistically explained at the level of capsid host cell interactions, where the genus departs from the classical *Paslahepevirus* paradigm: rat HEV virus-like particles (VLPs) bind human liver and intestinal cells and tissues with high efficiency and penetrate the cell membrane to enter target cells post-binding, whereas ferret HEV VLPs show only marginal binding and entry, and bat and avian HEV VLPs exhibit none (Guo et al., 2024). Structure-based three-dimensional mapping further identifies the surface spike domain of the rat HEV capsid as the determinant of this cell binding (Guo et al., 2024), indicating that capsid–receptor engagement rather than host physiology alone shapes the distinct tropism profile of this genus and that HEV-C1 achieves zoonotic infection through a species-specific capsid–receptor interface that is not shared with the entry repertoires of other viral members.

Notably, the divergence from *Paslahepevirus* is serologically incomplete: rat HEV partially cross-reacts with *Paslahepevirus*, and sera from patients infected with *Paslahepevirus* or from Hecolin^®^-vaccinated individuals partially cross-protect against rat HEV VLP binding to human target cells (Guo et al., 2024). This combination of efficient binding to liver and intestinal cells, a spike-domain-dependent entry strategy, and partial immune cross-reactivity positions *Rocahepevirus* as a second, genuinely systemic zoonotic pathway whose tropism must be weighed alongside *Paslahepevirus* in any complete account of HEV pathogenesis (Yadav et al., 2025).

These observations necessitate a qualification of the dual-module heuristic proposed in this review. That heuristic was constructed from entry studies of *Paslahepevirus*, in which a ubiquitous core module mediates basal entry and a liver-enriched ASGPR–EGFR module amplifies hepatocyte infection; rat HEV, however, engages human target cells through a spike-domain-dependent interaction that supports high-efficiency binding to both liver and intestinal cells, a tropism profile not readily explained by the HEV-centric (*Paslahepevirus*) core/amplifier architecture (Guo et al., 2024). The receptor network paradigm therefore requires species-specific adjustment: rather than a single genus-spanning entry network, the capsid surface of each lineage calibrates receptor engagement, reinforcing the view of HEV as a systemic pathogen while cautioning against *a priori* extrapolation of HEV (*Paslahepevirus*) entry data to *Rocahepevirus*. Whether HEV-C1 additionally engages the downstream core-module factors used by *Paslahepevirus*, such as ITGB1 or ASGPR, remains unknown; initial attachment to human hepatocytes has been shown to rely on heparan sulfate proteoglycans (Guo et al., 2024), so the completeness of the core/amplifier network in *Rocahepevirus* warrants further investigations.

### 3.1 Virus structure and genome organization

HEV is a small RNA virus with icosahedral morphology and a diameter of 27–34 nm. It is shed into bile and feces as a non-enveloped (naked) particle but circulates in blood and cell culture supernatants as a quasi-enveloped virion cloaked in a host-derived lipid membrane (Nagashima et al., 2017; Wang and Meng, 2021). The biological and entry-relevant distinctions between these two virion forms are discussed in detail in section “4.1 Host receptor networking during HEV entry.” Its genome consists of approximately 7.2 kb of single-stranded, positive-sense RNA and contains three major open reading frames (ORF1, ORF2, and ORF3) (Tam et al., 1991; Ahmad et al., 2011; Kenney and Meng, 2019; Figure 3). ORF1 encodes a large non-structural polyprotein of approximately 186 kDa that is involved in viral genome replication and is annotated by sequence homology into putative functional regions, including a methyltransferase, a Y domain, a papain-like cysteine protease region, a hypervariable region, X domain, a helicase, and an RNA-dependent RNA polymerase (Sehgal et al., 2006; Holla et al., 2013; Kenney and Meng, 2019). It must be emphasized, however, that this domain organization reflects sequence-based annotation rather than experimentally validated processing. In the classical model, patterned after other positive-sense RNA viruses, the ORF1 polyprotein is cleaved by its own PCP into discrete functional units (Nan and Zhang, 2016). Whether pORF1 is ever proteolytically processed in the context of authentic infection remains one of the most contentious questions in HEV molecular virology (Karpe, 2024). Studies in heterologous expression systems have yielded conflicting results, with some reporting cleavage and others reporting none, and full-length pORF1 generally remaining the abundant species (Nan and Zhang, 2016). Recent structural work has sharpened this skepticism: the crystal structure of a genotype 1 pORF1 fragment (residues 510–691) overlapping the C-terminal portion of the putative PCP region (residues 434–783) provided no support for protease function, leading Fieulaine and colleagues to argue that the terms “HEV protease” and “PCP domain” should be abandoned altogether (Fieulaine et al., 2023a,b). Consistent with this monomeric view, ORF1 has been shown to operate as a multifunctional protein that is not subject to proteolytic processing, in which the pPCP region functions structurally rather than catalytically six of its cysteines are essential for replication through divalent metal ion coordination that stabilizes local and interdomain interactions, and the region can rescue replication only within full-length ORF1, not as an individual subdomain (LeDesma et al., 2023). A third, intermediate model holds that processing, when it occurs, is mediated not by a viral protease but by host proteases: thrombin cleaves the HEV polyprotein at multiple conserved locations, and this cleavage is required for genome replication (Pierce et al., 2023). A recent review distills the field into four non-exclusive possibilities: pORF1 may not be processed at all and function as a multifunctional polyprotein; pORF1 may lack intrinsic protease activity; host proteases may contribute to processing; or processing may occur only to a limited extent in a specific cellular microenvironment (Karpe, 2024). This controversy is directly relevant to the present review: subsequent sections attribute innate immune-evasion functions to defined ORF1 regions most prominently, methyltransferase-mediated inhibition of type I interferon induction via targeting of RIG-I (Nan et al., 2014b; Kang et al., 2018), a property of ORF1 products demonstrated independently of the processing question (Nan et al., 2014b; LeDesma et al., 2023). Given that the monomeric-polyprotein model now holds comparable and arguably greater experimental support in light of recent structural work, such functions should be read as region-level activities of the ORF1 polyprotein whether liberated by cleavage or embedded within it rather than as established functions of independently processed domains.

**FIGURE 3:**
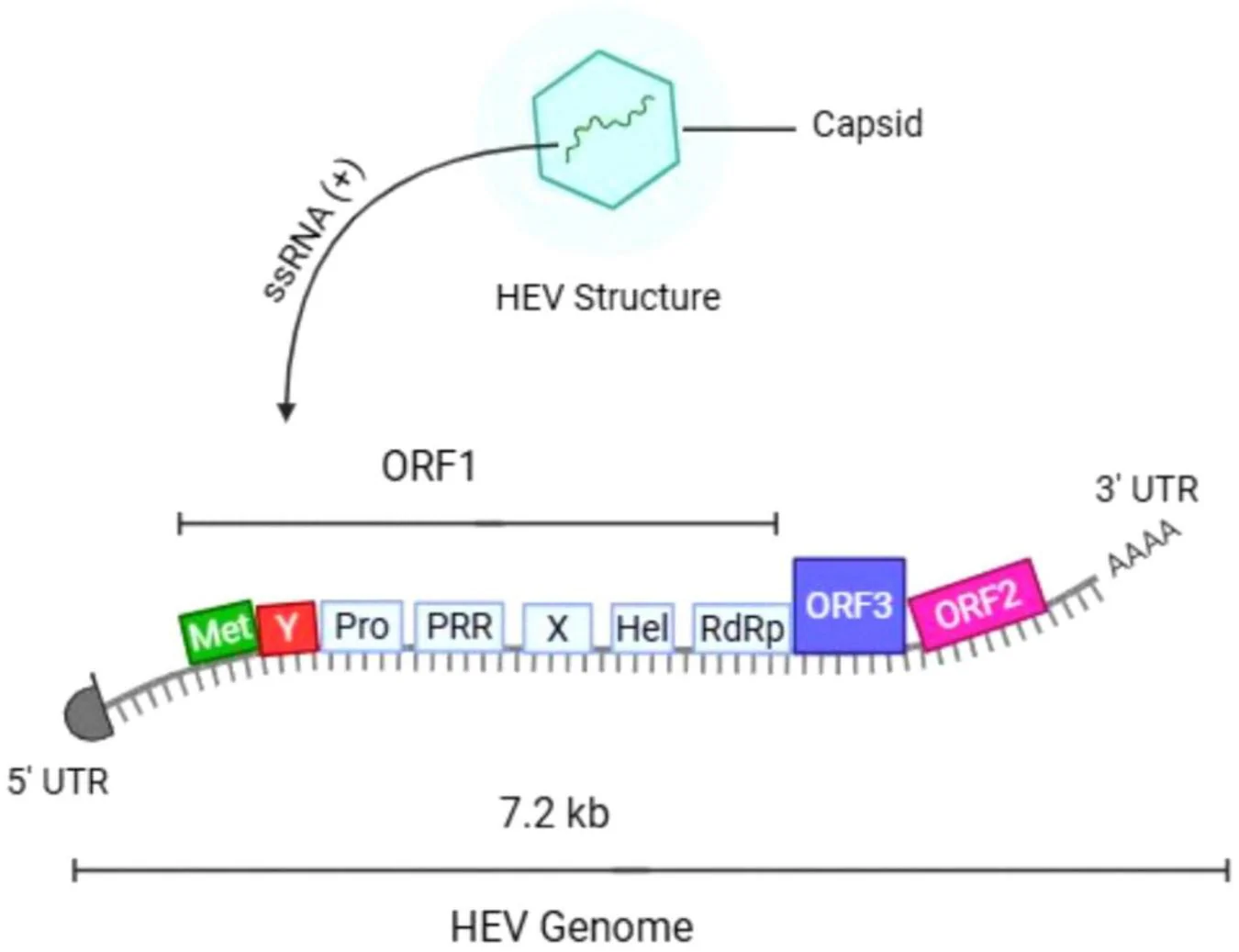
Schematic illustration of the hepatitis E virus (HEV) virion structure and genome organization. *ORF1 processing into discrete units vs. a single multifunctional protein remains debated.

ORF2 encodes a capsid protein that forms the viral shell, while ORF3 encodes a multifunctional phosphoprotein that modulates host pathways and aids virion release (Table 2; LeDesma et al., 2019). In addition to these three conserved ORFs, genotype 1 HEV encodes a fourth, genotype-specific reading frame (ORF4), embedded within ORF1 in an alternate frame. ORF4 is translated via an IRES-like element activated by endoplasmic reticulum stress and forms a replication complex with the viral helicase, RdRp, and host eEF1α1 that enhances RdRp activity, thereby promoting genotype-1-specific replication (Nair et al., 2016). The genome also contains 5′ and 3′ untranslated regions (UTRs) as well as cis-acting elements that facilitate RNA stability, replication, and translation. These characteristics create a genome architecture that enables efficient viral replication and evasion of host immune defenses (Nan et al., 2014b; Yin et al., 2018; Ju and Ding, 2019; Wang and Meng, 2021).

**TABLE 2:** HEV proteins and their role in replication.

ORF	Function	Role in replication	References
ORF1	Replication of viral genome	Drives replication and synthesis of viral RNA through its enzymatic activities (putative methyltransferase, putative helicase, putative RdRp)	Nan and Zhang, 2016; Kenney and Meng, 2019
ORF2	Capsid formation	Forms the viral shell that packages the genome (as secreted ORF2S, it modulates humoral immunity)	Nagashima et al., 2017; Yin et al., 2018; Wang and Meng, 2021
ORF3	Viral release, immune modulation	Aids release of quasi-enveloped virions via the ESCRT/exosomal pathway and modulates host immune responses (e.g., TLR7 suppression)	Nagashima et al., 2011; Lei et al., 2018
ORF4	ER-stress-induced enhancement of replication	Stimulates RdRp activity via a replication complex with helicase, RdRp, and host eEF1α1	Nair et al., 2016; Kenney and Meng, 2019

## 4 HEV pathogenesis

Hepatitis E is the inflammation of the liver caused by the hepatitis E virus. HEV infection is recognized as a leading cause of acute hepatitis. Beyond its hepatic manifestations, HEV has demonstrated a capacity to infiltrate and replicate within tissues far removed from the liver, raising important questions about the mechanisms that govern its broader tissue tropism. The disease can also progress to a chronic phase and manifest as systemic extrahepatic complications. These extrahepatic manifestations include neurological disorders, renal impairment, and pancreatitis, demonstrating that HEV possesses an expanded tissue tropism (Devhare et al., 2013; Kamar et al., 2016; Fousekis et al., 2019; El-Mokhtar and Sayed, 2021; Yadav and Kenney, 2023). Critically, this expanded tropism is not uniform across the *Hepeviridae*: the emerging zoonotic rat HEV (*Rocahepevirus ratti*) departs from the classical *Paslahepevirus* paradigm through lineage-specific capsid–receptor interactions, with rat HEV virus-like particles binding human liver and intestinal cells and tissues with high efficiency via a surface spike-domain capsid determinant (Guo et al., 2024). Such lineage-specific receptor engagement complicates any “one-size-fits-all” model of HEV tropism and zoonosis, indicating that tissue permissiveness and cross-species potential must be defined for individual viral lineages rather than extrapolated from the *Paslahepevirus* paradigm (Guo et al., 2024). The underlying mechanisms facilitating these extrahepatic sequelae remain a subject of active investigation. These observations indicate a broader physiological vulnerability to HEV infection than previously understood and necessitate the characterization of the receptor profiles that govern viral entry in non-hepatic tissues. Any such characterization should build on the foundational finding that HEV capsid binding is initiated by low-affinity electrostatic interactions between the ORF2 protein and cell surface heparan sulfate proteoglycans, particularly syndecans, whose removal markedly reduces ORF2 binding and viral infection (Kalia et al., 2009; Alkan et al., 2025). Elucidating the mechanisms that permit viral entry and replication across diverse organ systems remains a pivotal area of research for the development of targeted therapeutics and interventions to mitigate viral pathogenesis.

### 4.1 Host receptor networking during HEV entry

#### 4.1.1 Current concepts of HEV cell entry

HEV cell entry is a stepwise process that remains incompletely understood. Understanding the mechanisms governing HEV cell entry is critical because these mechanisms influence the broad tissue tropism, multi-host range, and capacity of HEV to produce both acute and chronic disease (Brüggemann et al., 2025; Fu et al., 2025). HEV exists in two distinct virion forms: non-enveloped (naked) virions (nHEV), shed into bile and feces and playing a central role in inter-host transmission via the fecal-oral route, and quasi-enveloped virions (eHEV), cloaked in a host-derived lipid membrane and circulating in the blood to facilitate intra-host dissemination, including cell-to-cell spread. Both forms are infectious but utilize distinct attachment factors and follow divergent intracellular trafficking routes, reflecting the fact that HEV cell entry is not a single linear event but a branched process determined by the biophysical form of the virion (Fu et al., 2025).

Initial binding involves interactions between the HEV capsid and glycans or broadly distributed molecules on the plasma membrane, followed by engagement of one or more entry-associated factors that confer higher specificity (Kalia et al., 2009; Brüggemann et al., 2025). In the case of eHEV, interactions with phosphatidylserine exposed on the quasi-envelope surface contribute an additional layer of specificity (Corneillie et al., 2023). Unlike HCV, for which essential entry receptors have been clearly defined (CD81, CLDN1, and OCLN) (Gerold et al., 2020), no single high-affinity HEV receptor has been definitively identified. Instead, HEV entry is governed by a network of host cell surface molecules acting in concert (Brown et al., 2025; Brüggemann et al., 2025), leading to the concept of a receptor network or portfolio rather than a single-receptor paradigm. The host factors implicated in this network, their virion-form specificity, and the strength of the experimental evidence supporting each are summarized in Table 3 and illustrated in Figure 4.

**TABLE 3:** Host cell factors and trafficking machinery implicated in HEV infection.

Host factor	Functional role	Virion form	Step	Key reference(s)
A. Entry-associated factors				
HSPGs (Syndecans)	Low-affinity electrostatic attachment factor; infection reduced by heparinase treatment. 6-O-sulfation-dependent syndecan binding established by VLP studies.	nHEV	Attachment	Kalia et al., 2009; Brüggemann et al., 2025
ASGPR1/2	Hepatocyte-enriched attachment lectin; directly engages pORF2; mainly regulates viral binding and entry steps.	nHEV	Viral binding and entry	Zhang et al., 2016; Brown et al., 2025; Brüggemann et al., 2025
ITGB1 (Integrin β1)	Key entry cofactor; pairs with different α-integrins to mediate nHEV cell entry; directs nHEV into Rab11+ recycling endosomes.	nHEV	Internalization	Brüggemann et al., 2025; Fu et al., 2025
TIM1/HAVCR1	PS-binding entry cofactor; facilitates eHEV internalization via interaction with phosphatidylserine in the quasi-envelope.	eHEV	Internalization	Corneillie et al., 2023
B. Trafficking machinery				
Clathrin/dynamin endocytosis	Primary internalization route for both virion forms.	nHEV and eHEV	Internalization	Kapur et al., 2012; Holla et al., 2015; Yin et al., 2016
Rab11+ recycling endosome	nHEV-specific trafficking route downstream of ITGB1 engagement.	nHEV	Endosomal trafficking	Fu et al., 2025
Rab5a+ early endosome	eHEV-specific early endosomal trafficking route.	eHEV	Endosomal trafficking	Yin et al., 2016; Brüggemann et al., 2025; Fu et al., 2025
Rab7+ late endosome	Late endosomal trafficking route toward lysosomal compartments.	nHEV and eHEV	Endosomal trafficking	Yin et al., 2016; Brüggemann et al., 2025; Fu et al., 2025
C. Lysosomal processing factors				
NPC1	Required for eHEV quasi-envelope degradation alongside LAL; NPC1 depletion reduces eHEV infection by ∼50%, without substantially impacting overall HEV infectivity.	eHEV	Lysosomal processing	Yin et al., 2016; Ahmad et al., 2023; Brüggemann et al., 2025
Cathepsin L (CTSL)	Principal lysosomal protease for capsid proteolysis and genome release.	nHEV and eHEV	Uncoating and genome release	Klöhn et al., 2024; Fu et al., 2025
D. Candidate and indirect host factors				
EGFR	Candidate entry cofactor via the extracellular ligand-binding domain; exerts a stage-dependent antiviral function at the replication and release stages, where EGFR inhibition enhances replication and exosomal release.	nHEV and eHEV	Entry mechanism undefined	Schrader et al., 2023; Woytinek et al., 2024; Brüggemann et al., 2025
GRP78 (BiP)	Candidate attachment cofactor; binds p239 in ATP-sensitive manner; blocks p239 attachment to HepG2 cells.	Not determined	Attachment	Xiao-cheng et al., 2007; Zheng et al., 2010
HSC70	Candidate cofactor; proposed role in intracellular transport or HEV replication.	Not determined	Not established	Kenney and Meng, 2019
ATP5B	Candidate entry cofactor; identified by VLP-based pull-down/mass spectrometry; validated by antibody and siRNA-mediated approaches and infectious HEV from the stools of a hepatitis E patient.	nHEV	Entry mechanism undefined	Ahmed et al., 2016

nHEV, non-enveloped HEV; eHEV, quasi-enveloped HEV; HSPGs, heparan sulphate proteoglycans; ASGPR1/2, asialoglycoprotein receptor 1/2; ITGB1, integrin β1; TIM1/HAVCR1, T cell immunoglobulin and mucin domain-1; PS, phosphatidylserine; NPC1, Niemann-Pick C1 protein; LAL, lysosomal acid lipase; CTSL, cathepsin L; EGFR, epidermal growth factor receptor; GRP78, glucose-regulated protein 78; HSC70, heat shock cognate 70 kDa protein; ATP5B, ATP synthase subunit β; MS, mass spectrometry.

**FIGURE 4:**
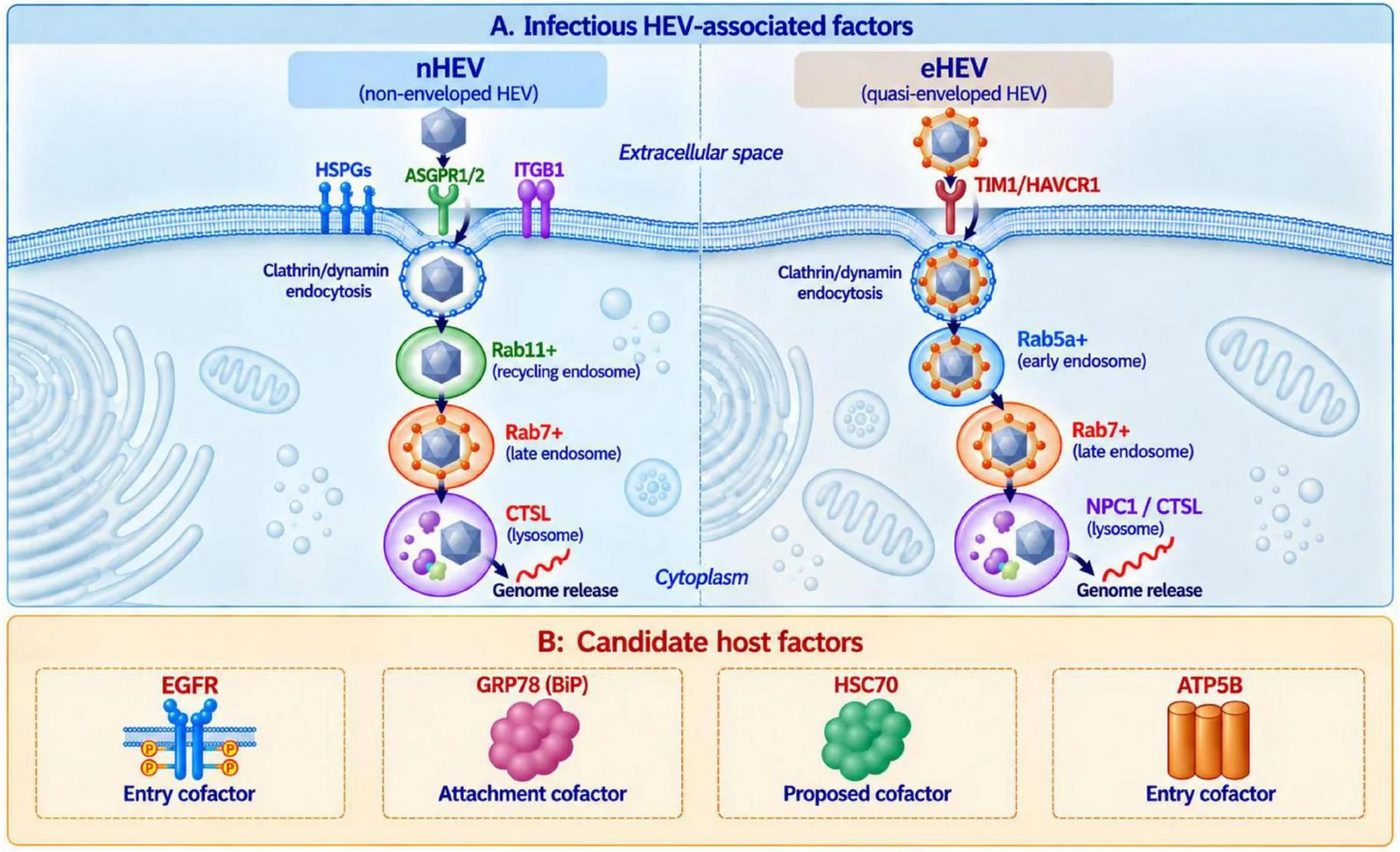
Evidence-stratified entry pathways and host factors of HEV. HEV exists as two virion forms with distinct entry routes. Non-enveloped HEV (nHEV) attaches via HSPGs and ASGPR1/2, with ITGB1 acting as an entry cofactor directing clathrin/dynamin-mediated endocytosis into Rab11+ recycling endosomes, followed by transit through Rab7+ late endosomes and CTSL-mediated lysosomal processing to genome release. Quasi-enveloped HEV (eHEV) is internalized via TIM1/HAVCR1 interaction with phosphatidylserine in the quasi-envelope, then traffics through clathrin/dynamin-mediated endocytosis into Rab5a+ early endosomes, Rab7+ late endosomes, and NPC1-dependent lysosomal processing with CTSL-mediated genome release. Candidate host factors (EGFR, GRP78, HSC70 and ATP5B) are shown separately. nHEV, non-enveloped HEV; eHEV, quasi-enveloped HEV; HSPGs, heparan sulphate proteoglycans; ASGPR1/2, asialoglycoprotein receptor 1/2; ITGB1, integrin β1; TIM1/HAVCR1, T cell immunoglobulin and mucin domain-1; Rab5a, Ras-related protein 5a; Rab7, Ras-related protein 7; Rab11, Ras-related protein 11; NPC1, Niemann-Pick C1 protein; CTSL, cathepsin L; EGFR, epidermal growth factor receptor; GRP78, glucose-regulated protein 78; HSC70, heat shock cognate 70 kDa protein; ATP5B, ATP synthase subunit β.

### 4.2 Attachment versus functional receptors

A critical distinction in HEV cell biology is between molecules that mediate initial surface attachment and those that serve as functional entry receptors or cofactors. Several molecules initially designated as HEV receptors are now understood to function primarily as attachment factors, while others facilitate post-binding steps including internalization and endosomal processing (Kalia et al., 2009; Brüggemann et al., 2025). Evidence supporting each factor varies considerably, as reflected in Figure 4 and Table 3.

#### 4.2.1 Heparan sulfate proteoglycans (HSPGs)

HSPGs, especially the syndecan family, were the first host cell surface molecules identified as binding factors for the HEV capsid. Kalia and colleagues demonstrated that recombinant ORF2 virus-like particles bind syndecans via defined 6-O-sulfation patterns, establishing the binding mechanism using VLP studies. They also demonstrated that heparinase treatment of cells significantly reduced HEV infection, providing evidence from infectious HEV for HSPG involvement (Kalia et al., 2009). This binding is not entirely due to electrostatic interactions. eHEV does not engage HSPGs due to its membrane encasement and the shielding of the capsid surface by the quasi-envelope (Brüggemann et al., 2025).

#### 4.2.2 Asialoglycoprotein receptor (ASGPR)

ASGPR is a heterodimeric C-type lectin expressed on the basolateral surface of hepatocytes that binds glycoproteins bearing exposed terminal galactose residues (Kumar and Turnbull, 2023). Within the receptor portfolio, this lectin operates at a defined step downstream of the initial capsid–cell encounter: the foundational work of Kalia and colleagues established that cell-surface heparan sulfate proteoglycans, specifically syndecans, are required for pORF2 binding to liver cells, with enzymatic (heparinase) or chemical (sodium chlorate) removal of cell-surface heparan sulfate causing a marked reduction in capsid binding, and heparinase treatment substantially reducing HEV infection (Kalia et al., 2009). Zhang and colleagues demonstrated that the HEV ORF2 ectodomain directly interacts with both ASGR1 and ASGR2, that susceptibility to HEV infection correlates with ASGPR expression across different cell lines, and that ASGPR ligands competitively inhibit HEV binding in a calcium-dependent manner (Zhang et al., 2016). This sequential organization HSPG-mediated attachment followed by ASGPR-mediated facilitation maps directly onto the dual-module heuristic. HSPGs constitute the ubiquitous core module mediating basal attachment across nucleated cells, whereas ASGPR represents the liver-enriched amplifier that concentrates and facilitates entry at the hepatocyte surface. As ASGPR is restricted to the basolateral membrane of polarized hepatocytes, it contributes to hepatotropism by restricting productive infection to cells accessible at the sinusoidal surface. However, ASGPR should be understood as a hepatocyte-enriched entry-associated attachment factor rather than as an essential canonical receptor, since available studies demonstrate facilitation of binding and infection rather than an obligate gating function (Zhang et al., 2016; Brown et al., 2025; Brüggemann et al., 2025).

#### 4.2.3 Integrins

Integrins are heterodimeric transmembrane receptors linking the extracellular matrix to the cytoskeleton. Although integrin α3 (ITGA3) was previously proposed as an HEV host factor, a recent high-content imaging study by Fu and colleagues demonstrated that ITGA3 is not expressed in the most HEV-permissive cell types and is therefore not a functional entry receptor (Fu et al., 2025). Instead, integrin β1 (ITGB1), paired with various α integrins, was identified as a cofactor mediating nHEV internalization. ITGB1 engagement channels nHEV into Rab11+ recycling endosomes, a route that was directly demonstrated with infectious nHEV and is distinct from the classical early endosomal pathway (Fu et al., 2025). eHEV virions do not interact with ITGB1 and, after clathrin/dynamin-mediated uptake (shared with nHEV), traffic through Rab5a+ early endosomes and thence to Rab7+ late endosomes toward lysosomal processing (Yin et al., 2016; Ahmad et al., 2023; Fu et al., 2025). This divergence in intracellular trafficking demonstrates that nHEV and eHEV use fundamentally distinct post-internalization routes.

#### 4.2.4 TIM1/HAVCR1

TIM1 is a phosphatidylserine-binding receptor of the T cell immunoglobulin and mucin family that facilitates eHEV internalization through direct interaction with phosphatidylserine exposed on the outer leaflet of the quasi-envelope (Corneillie et al., 2023). Corneillie and colleagues demonstrated, using CRISPR knockout, rescue experiments, ectopic expression, phosphatidylserine-binding-pocket mutagenesis, and Annexin V inhibition with eHEV infection assays, that TIM1 promotes eHEV but not nHEV cell entry (Corneillie et al., 2023). Notably, TIM1 does not affect viral attachment: no difference in viral binding was observed between wild-type and TIM1-knockout cells, whereas internalization assays showed an almost 1-log decrease in internalized eHEV in TIM1-knockout cells relative to wild-type cells, with internalized nHEV unchanged (Corneillie et al., 2023). TIM1 therefore acts specifically at the internalization step rather than as an attachment factor, engaging the virion membrane rather than the capsid (Corneillie et al., 2023). This positions TIM1 as an eHEV-specific entry cofactor that completes the divergent entry-route argument: while nHEV entry is governed by capsid–receptor interactions HSPG-mediated attachment followed by ASGPR facilitation eHEV entry proceeds through phosphatidylserine-mediated apoptotic mimicry at the level of the quasi-envelope, a mechanism absent from the naked virion pathway (Corneillie et al., 2023).

#### 4.2.5 NPC1, lysosomal acid lipase, and cathepsin L

Following internalization, both nHEV and eHEV require lysosomal processing for productive uncoating and genome release. For eHEV, the quasi-envelope must be disassembled within the lysosome prior to capsid exposure (Brüggemann et al., 2025). Yin and colleagues demonstrated that eHEV infectivity is significantly reduced in NPC1-depleted cells and in cells treated with a lysosomal acid lipase inhibitor, establishing NPC1 and lysosomal acid lipase (LAL) as required factors for endolysosomal membrane remodeling during eHEV entry (Yin et al., 2016; Ahmad et al., 2023). NPC1 is not a direct genome translocator; its precise molecular mechanism of action remains to be fully defined. Lysosomal cathepsins, particularly cathepsin L (CTSL), mediate proteolytic processing of both virion forms, which is required for productive uncoating and genome release (Brüggemann et al., 2025). Klöhn and colleagues demonstrated that cathepsin L is critical for HEV infection, identifying CTSL as a critical lysosomal protease in this step (Feng, 2024; Klöhn et al., 2024; Fu et al., 2025).

#### 4.2.6 Epidermal growth factor receptor (EGFR)

Epidermal growth factor receptor (EGFR) is a receptor tyrosine kinase co-opted for cell entry by other hepatotropic viruses, including HCV, where it facilitates virion internalization and activates the RAF–MEK–ERK signaling pathway that regulates sorting and maturation of internalized virions toward the site of uncoating (Schrader et al., 2023; So et al., 2023). Its role in the HEV life cycle, however, is not uniform: EGFR acts as a proviral factor during entry but exerts an antiviral influence during the later replication and release stages, and delineating these phases is essential for correctly situating EGFR within the dual-module heuristic. Woytinek and colleagues demonstrated that during HEV infection, significant downregulation of EGFR mRNA and protein was observed, and that this downregulation was reversed by ribavirin treatment, indicating that it is directly driven by viral infection (Woytinek et al., 2024).

During the entry phase, EGFR functions as a proviral entry cofactor. Silencing of EGFR reduces initial HEV infection events by blocking viral entry (Schrader et al., 2023); EGFR involvement appears to operate via its extracellular ligand-binding domain rather than its kinase or signaling function and has been documented in the context of both nHEV and eHEV (Schrader et al., 2023; Brüggemann et al., 2025). Consistent with its role as a cofactor rather than a receptor, EGFR is engaged after viral attachment, and it has been proposed either to provide ligand-binding-dependent endosomal trafficking routes exploited by the virus or to stabilize the viral entry receptor complex (Brüggemann et al., 2025). At the replication and release stages, the direction of this effect reverses, and here EGFR functions as an antiviral host factor. Suppression of EGFR by siRNA-mediated silencing or by pharmacological inhibition with the tyrosine kinase inhibitors erlotinib and gefitinib significantly increased extracellular viral titers, and erlotinib in particular enhanced replication, elevating both intracellular and extracellular viral RNA transcripts and capsid protein across different genotype 3 strains and cell types, including liver-derived PLC/PRF/5 cells (Woytinek et al., 2024). Precisely because inhibiting EGFR kinase signaling at later stages enhances viral replication and release, EGFR itself is antiviral at this stage: its active signaling restrains viral propagation, at least in part by sustaining innate antiviral programs through the Src–JAK–STAT axis, and EGFR downregulation or inhibition removes this brake, promoting viral replication, exosome-mediated release, and evasion of innate immune responses (Woytinek et al., 2024). Mechanistically, EGFR inhibition increased expression of the multivesicular body marker CD63, induced autophagy in a pattern mirroring the kinome profile of HEV-infected cells, and caused lysosomal cholesterol accumulation that reduced cholesterol levels outside lysosomes, collectively favoring viral release via the exosomal route (Woytinek et al., 2024). Notably, while EGFR inhibition also impaired initial viral binding and entry, likely reflecting partial internalization of the receptor, this anti-entry effect was outweighed by the proviral consequences at the later stages (Woytinek et al., 2024). In this dual-phase model, EGFR plays contrasting roles: it aids viral entry as part of the liver-specific ASGPR–EGFR complex, but acts as an antiviral defense factor during viral replication and release, prompting the virus to actively downregulate it. (Woytinek et al., 2024) shifts the balance toward replication, egress, and immune escape. EGFR’s role as a direct entry receptor has not been established, and its precise mechanistic role remains unresolved (Brüggemann et al., 2025). EGFR is therefore classified as a candidate entry cofactor rather than an established entry receptor.

#### 4.2.7 Glucose regulated protein 78 (GRP78/BiP) and heat shock cognate protein 70 (HSC70)

GRP78/BiP has been shown to interact with the HEV p239 capsid protein in an ATP-sensitive, conformation-dependent manner, and preincubation with GRP78/BiP significantly blocked p239 attachment to HepG2 cells, suggesting a role at the attachment step (Xiao-cheng et al., 2007). HSC70 has also been proposed as a candidate cofactor with a role in intracellular transport or HEV replication, although the evidence derives exclusively from recombinant capsid binding studies (Kenney and Meng, 2019). Neither GRP78 nor HSC70 has been validated in infectious virus entry assays, and both are therefore classified as indirect candidate factors.

#### 4.2.8 ATP synthase subunit β (ATP5B)

Mitochondrial F_1_F_0_-ATP synthase subunit β is detectable on the plasma membrane of many cell types and has been proposed to facilitate the entry of several viruses (Ueda and Suwanmanee, 2022; Chang et al., 2023). As discussed above, capsid binding depends on cell-surface heparan sulfate proteoglycans, particularly syndecans. Building on this attachment paradigm, Ahmed and colleagues identified ATP5B as an HEV capsid-interacting protein by VLP-based pull-down and mass spectrometry, using HEV-LPs expressed in *E. coli*, and subsequently validated this interaction using antibody- and siRNA-mediated approaches with infectious HEV from the stool of a hepatitis E patient (Ahmed et al., 2016). ATP5B thereby joined a growing portfolio of capsid-interacting host factors, including GRP78, α-tubulin, HSP90, cytochrome P450 2C8, HSPGs, and integrin α3, identified through truncated-capsid screens (Fan et al., 2021). ATP5B is therefore classified as a candidate entry cofactor, while its precise mechanistic role in productive entry remains undefined.

Since this study, higher-resolution functional work has substantially raised the validation bar for HEV entry factors, most notably for integrin β1. As discussed earlier, ITGB1 has been supported by structural modeling of the capsid–ITGB1 interface, proximity ligation confirmation, knockout abolition, and D522E impairment, whereas ATP5B lacks these additional lines of evidence.

Consistent with this, the most recent mechanistic inventories of nHEV attachment factors emphasize ASGPR1/2, HSPGs, and integrins rather than ATP5B (Brüggemann et al., 2025). ATP5B should therefore be retained as an interaction-validated but mechanistically uncharacterized candidate entry cofactor whose validation status falls below that of ITGB1; whether it contributes to the ubiquitous core module or the liver-enriched amplifier of the dual-module heuristic remains an open question.

### 4.3 Receptor networks and HEV tissue tropism

#### 4.3.1 Hepatic tropism

Hepatitis E virus (HEV) can infect many types of cells; however, hepatocytes remain its principal target. Current evidence suggests that HEV hepatotropism involves a combination of host-cell entry factors and hepatocyte-specific cellular features rather than dependence on a single receptor, consistent with the portfolio of attachment factors ASGPR1/2, HSPGs, and integrins described for nHEV (Brüggemann et al., 2025).

Three classes of hepatocyte-enriched molecules contribute to this preferential tropism. First, the asialoglycoprotein receptor directly interacts with the HEV ORF2 ectodomain, engaging both ASGR1 and ASGR2, and susceptibility to HEV correlates positively with surface ASGPR expression across cell lines (Zhang et al., 2016). ASGPR-directed siRNA does not affect viral release, localizing ASGPR function to the binding and entry steps, and classic ASGPR ligands competitively inhibit HEV binding in the presence of calcium; consistently, ectopic ASGPR expression increases HEV-binding capacity while ASGPR knockout reduces it (Zhang et al., 2016). Second, EGFR is engaged as a candidate entry cofactor after viral attachment, an involvement that does not rely on its kinase or signaling function but rather on its extracellular ligand-binding domain and that has been documented for both nHEV and eHEV (Schrader et al., 2023; Brüggemann et al., 2025); silencing EGFR reduces initial HEV infection events by blocking viral entry (Schrader et al., 2023). EGFR’s role, however, is stage-dependent. HEV infection actively downregulates EGFR mRNA and protein, and EGFR suppression by siRNA or by the tyrosine kinase inhibitors erlotinib and gefitinib significantly increases extracellular viral titers. Erlotinib, in particular, enhanced replication and elevated both intracellular and extracellular viral RNA transcripts and capsid protein across genotype 3 strains and cell types, indicating that sustained EGFR engagement exerts an antiviral function at the later replication and release stages, at least in part through the Src–JAK–STAT axis (Woytinek et al., 2024). Notably, both EGFR and ASGPR are primarily restricted to the basolateral membrane of polarized cells, the membrane domain through which hepatotropic viruses HAV, HBV, and HCV among them infect highly polarized hepatocytes (Brown et al., 2025); this localization is consistent with a hepatocyte-entry role for the ASGPR–EGFR module and with the demonstration that polarized hepatoma models, which recapitulate hepatocyte polarity *in vivo*, support HEV infection with clinical isolates (Brown et al., 2025). Third, integrin β1, pairing with exchangeable α-integrin partners, has been identified as the principal cofactor mediating nHEV cell entry, directing virions through Rab11-positive recycling endosomes. Hepatocytes are also enriched in HSPGs such as syndecans, which mediate initial low-affinity capsid attachment. Removal of cell-surface heparan sulfate by heparinase or chlorate, or competition with heparin and sulfated derivatives, markedly reduces pORF2 binding and HEV infection (Kalia et al., 2009). Taken together, the convergence of these hepatocyte-enriched factors at the surface of hepatic cells provides a plausible molecular basis for HEV hepatotropism, although the relative contribution of individual factors and post-entry cellular determinants has not been definitively established; which regions of the cell membrane are initially exposed to HEV, and the contribution of tight-junction constituents to viral attachment and entry, likewise remain open questions (Brown et al., 2025).

Viewed this way, the dual-module heuristic offers one plausible lens through which to organize this receptor portfolio. Within this framework, the ubiquitous core module comprises broadly expressed machinery shared by both virion forms, including HSPG-mediated capsid attachment, clathrin/dynamin-dependent endocytosis, Rab-governed endosomal trafficking downstream of ITGB1 (nHEV) or Rab5a (eHEV), and NPC1/cathepsin-dependent lysosomal processing (Kalia et al., 2009; Brüggemann et al., 2025; Fu et al., 2025). By contrast, the liver-enriched ASGPR–EGFR amplifier is engaged at the capsid level, ASGPR binds the nHEV capsid directly, and EGFR operates post-attachment for both forms, whereas eHEV diverts to an envelope-level entry route via phosphatidylserine–TIM1 engagement that bypasses the capsid-directed arm of the amplifier while still converging on the core lysosomal processing machinery (Zhang et al., 2016; Corneillie et al., 2023; Brüggemann et al., 2025). The heuristic should nevertheless be treated as provisional rather than established, and as a framework derived predominantly from *Paslahepevirus*, whose genus-level generality remains to be tested.

Indeed, this hepatocyte-centric account is derived from *Paslahepevirus* and does not seamlessly generalize across the genus. As detailed in the Genetic Diversity and Classification section, *Rocahepevirus ratti* (rat HEV) engages the cell surface through distinct capsid–receptor interactions. Rat HEV VLPs bind and enter human liver and intestinal cells and tissues with high efficiency, whereas ferret HEV VLPs show only marginal binding and entry, and bat and avian HEV VLPs exhibit none. Structure-based mapping further identifies the surface spike domain of the rat HEV capsid as the determinant of this cell binding (Guo et al., 2024). Together with the expanding documentation of rat HEV as a zoonotic pathogen spilling over from rodents into humans, affecting both immunocompetent and immunocompromised hosts (Yadav et al., 2025), this lineage-specific capsid–receptor engagement complicates any “one-size-fits-all” heuristic and reinforces the view of HEV tropism as complex and genus-dependent. Whether rat HEV additionally engages core-module factors such as HSPGs, ITGB1, or ASGPR remains unknown and is identified in the classification section as a research gap.

#### 4.3.2 Extrahepatic tropism

Hepatitis E virus (HEV) is increasingly recognized as capable of interacting with or being detected in a range of extrahepatic tissues, although the extent to which these findings reflect productive viral replication remains incompletely understood. This is consistent with the constellation of extrahepatic manifestations observed, especially in immunocompromised patients (Fousekis et al., 2019; Thakur et al., 2020; Yadav and Kenney, 2023; Schlienkamp et al., 2026). Several animal and *in vitro* models have been developed to investigate HEV infection in extrahepatic tissues, providing evidence for HEV RNA or antigen detection and, in some systems, viral replication outside the liver. HEV-associated extrahepatic complications pose serious challenges for viral diagnosis and treatment (Schlienkamp et al., 2026). However, it remains unclear whether these manifestations result from direct viral replication or immune-mediated effects (Yadav and Kenney, 2023; Schlienkamp et al., 2026). Importantly, cellular permissiveness to HEV should not be interpreted solely in terms of viral entry. Differences between tissues or host species may also arise from post-entry compatibility with viral replication and from cell-intrinsic antiviral responses.

The dual-module heuristic remains a useful organizational lens for HEV entry, but it must be applied with two qualifications. First, it is derived almost exclusively from *Paslahepevirus*, and its genus-level generality is untested; the framework is therefore stated once in full in the preceding section and is not restated here. As detailed above, *Rocahepevirus ratti* engages target cells through distinct capsid–receptor interactions, and together with the expanding documentation of rat HEV zoonotic spillover into immunocompetent and immunocompromised hosts, this lineage-specific engagement complicates any one-size-fits-all tropism model. Second, receptor function is stage-dependent: EGFR acts provirally as a post-attachment entry cofactor (Schrader et al., 2023), yet HEV-driven EGFR downregulation enhances replication, release, and immune evasion at later stages, indicating an antiviral modulatory role (Woytinek et al., 2024). The heuristic therefore captures the entry phase but not the post-entry determinants of tissue tropism.

Beyond the liver, cumulative evidence supports dissemination of HEV or HEV-derived material to the CNS, kidney, gastrointestinal tract, and placenta (Billam et al., 2008; Bose et al., 2014; Shi et al., 2016; Tian et al., 2019). A crucial analytical distinction is between passive viral spread and active local replication. Detection of negative-strand viral RNA the replication intermediate indicates genuine replicative activity in extrahepatic tissues (Williams et al., 2001) Negative-strand RNA has likewise been detected in the pig intestinal tract, colon, and lymph nodes and in the liver, kidney, small intestine, spleen, and stomach of rabbit models (Williams et al., 2001; Letafati et al., 2024) Recent work emphasizes that extrahepatic complications may arise from local replication, immune-mediated damage, or both (Schlienkamp et al., 2026). On current evidence, these sites cannot yet be designated as secondary reservoirs; however, the clinical stakes are real. Should any prove to sustain persistent infection, the management of chronic hepatitis E and the risk of relapse in immunocompromised patients would be substantially complicated.

HEV RNA and viral antigens have been detected within the kidneys of infected hosts (Kamar and Pischke, 2019; El-Mokhtar and Sayed, 2021; Luo et al., 2024). The dual-module heuristic outlined above pertains specifically to intra-host tissue tropism and receptor-level determinants of hepatic versus extrahepatic spread and is not intended to account for interspecies host range, which is governed by a distinct and only partially overlapping set of viral and host determinants.

#### 4.3.3 Zoonotic potential and cross-species transmission risk

HEV genotypes 3 and 4 account for the majority of zoonotic HEV transmissions. Their ability to infect a wide array of animal hosts creates a vast viral reservoir, which may increase the opportunities for interspecies transmission. Furthermore, the emergence of novel strains may expand the zoonotic landscape, raising the possibility that current diagnostic surveillance may underestimate the true extent of cross-species transmission events. Genotype 7 is linked to dromedary camels and genotype 8 to Bactrian camels, while genotypes 5 and 6 have been identified in wild boars in Japan (Sridhar et al., 2017; Virhuez-Mendoza et al., 2025). Continuous genomic surveillance is required to monitor the evolution of these animal-derived HEV variants and their potential for human adaptation.

Serological and molecular evidence indicates a broad host range, as HEV or related viruses have been detected in various other species, including rabbits, rodents, deer, and dromedary camels, highlighting a complex landscape of animal reservoirs that could contribute to cross-species transmission risk. Recent national surveillance in Japan further illustrates this complexity. HEV infection was detected predominantly in wild boars, with multiple HEV-3 and HEV-4 subtypes circulating across different regions, whereas evidence of infection in other surveyed wildlife species was limited or absent. The recovery of infectious HEV from wild boar feces further supports the potential for environmental contamination and zoonotic exposure, while differences among wildlife species highlight species-specific variation in reservoir competence (Virhuez-Mendoza et al., 2025).

Critically, this zoonotic landscape is no longer confined to *Paslahepevirus*. *Rocahepevirus ratti* (rat HEV) has emerged as a zoonotic agent, with documented spillover from rodents into humans producing acute hepatitis in immunocompetent individuals (Benavent et al., 2023; Yadav et al., 2025; Rivero-Juarez et al., 2026) and persistent hepatitis in solid-organ transplant recipients and other immunocompromised hosts (Sridhar et al., 2018). Understanding the molecular and cellular determinants underlying cross-species transmission requires consideration of viral entry alongside post-entry replication compatibility and host antiviral responses.

## 5 Host receptors and cross-species transmission

HEV is the prototypical example of a mosaic virus whose genotypes differ markedly in their host range despite a high degree of genomic similarity (Shukla et al., 2011; Primadharsini et al., 2021; Kinast et al., 2022; Tang et al., 2026). Consistent with the scope noted above, host range in this context is discussed independently of the dual-module tropism heuristic, since cross-species transmission is shaped primarily by post-entry compatibility and viral genetic determinants rather than receptor-level tissue tropism. HEV-3 and HEV-4 are the principal zoonotic genotypes.

Although HEV-3 and HEV-4 are zoonotic viruses with a broad host range including swine, deer, and rabbits and HEV-1 and HEV-2 are obligate human pathogens, this differential host range does not appear to be set at the level of attachment alone. The interaction of the nHEV capsid with the promiscuous ITGB1 subunit, which pairs with exchangeable α-integrin partners, has been proposed to explain the broad tissue and species tropism of the zoonotic strain, with the human-restricted HEV-1/HEV-2 genotypes identified as the necessary future comparator (Brüggemann et al., 2025; Dufloo et al., 2025; Fu et al., 2025). Rather, restrictive barriers map to the capsid P domain. A chimeric HEV-3 virus bearing the HEV-1 P domain fails to infect swine cells, ORF2 proteins of genotypes 1 and 3 differ by 54 amino acids (8.2%), and HEV VLPs bind and enter cells in a species-specific manner, indicating that viral entry is a crucial determinant of host tropism and zoonotic potential. The *Rocahepevirus* example extends this principle to the capsid–receptor interface itself. Rat HEV VLPs bind and enter human liver and intestinal cells efficiently through the capsid spike domain, whereas ferret, bat, and avian VLPs show only marginal or no binding, implying entry determinants distinct from those captured by the *Paslahepevirus*-derived model (Zhou et al., 2021; Guo et al., 2024; Brüggemann et al., 2025).

The differential host ranges among these lineages make cross-species transmission a tractable mechanistic problem. Since HEV-3 and HEV-4, HEV-7, and rat HEV-C1 share a common genomic architecture yet differ markedly in host range, viral determinants, host-cell entry factors, post-entry replication compatibility, and antiviral responses can each be interrogated against a defined difference in host competence (Fu et al., 2025; Kinast et al., 2022; Primadharsini et al., 2021). The question this section addresses is therefore not which animal species harbor HEV, a landscape reviewed above, but which molecular barriers determine whether infection becomes productive in a new host.

Several studies have provided evidence for cross-species transmission. Existing literature indicates that two mechanistic classes of viral determinants have been linked to HEV host range. The first involves the ORF1 hypervariable region, which plays a role in host range and cellular tropism, although whether ORF1 acts as a processed polyprotein or a single multifunctional protein remains debated (Karpe, 2024; Pierce et al., 2023). The second involves ORF2 P-domain surface residues (Shukla et al., 2011; Meng, 2016; Nan et al., 2017; Tang et al., 2026). Chatterjee and colleagues reported that a chimeric HEV containing the ORF1 gene of HEV-4 in the backbone of HEV-1 can infect a pig kidney cell line, which supports a role for ORF1 in overcoming post-entry cellular restrictions associated with the HEV species barrier, although the study does not establish a role for ORF1 in viral entry or receptor usage (Chatterjee et al., 2016). The cell culture-adapted HEV-3 strain Kernow-C1/p6 contains a 171-nucleotide insertion derived from the human ribosomal protein S17 (RPS17) gene in ORF1 and displays an expanded *in vitro* host-cell range, including cell lines derived from cows, dogs, cats, chickens, and hamsters (Shukla et al., 2011; Kenney and Meng, 2015; Nan and Zhang, 2016; Fu et al., 2019; Wißing et al., 2024). Recent studies have demonstrated that HEV-1 replicates more efficiently than HEV-3 at the human maternal-fetal interface, consistent with the genotype-specific clinical severity of HEV-1 disease (Gouilly et al., 2018). Brüggemann and colleagues have suggested that the receptor portfolio supporting HEV entry may be broadly conserved across vertebrate cells, implying that species barriers may not be explained by the absence of a single essential receptor and may instead involve differences in cell-surface organization, entry pathways, endocytic routing, and post-entry cellular compatibility (Brüggemann et al., 2025). However, further studies are required to understand the cross-species transmission of HEV genotypes.

ORF2 P-domain residues 490 and 492 play an important role in host range restriction of HEV. Tang and colleagues used mAb 6H8, an antibody with selective binding ability to zoonotic HEV, to identify underlying mechanisms of host tropism. They identified an epitope involving residues 490 and 492 that plays a critical role in mAb 6H8 binding and in the attachment of the virus to host cells. However, the study concluded that these residues are important but not the sole determinant of HEV host tropism (Tang et al., 2026).

Overall, the species barrier is determined not solely by viral genetics or host determinants but by a complex array of interactions among viral genetic factors, host cellular factors, and immune status. This may also help explain why immunocompromised individuals are disproportionately susceptible to zoonotic HEV infections. From a receptor network standpoint, it is noteworthy that a viral genotype can bind to the broadly expressed HSPG/ITGB1/NPC1 core network but may cause disease only when the host immune system fails to clear the infection promptly. This could lead to replication of virions in non-hepatic cells, producing extrahepatic manifestations (Kinast et al., 2022; Ahmad et al., 2023; Yadav and Kenney, 2023; Fu et al., 2025; Schlienkamp et al., 2026).

Taken together, available *in vitro*, polarized-cell, and animal-model studies suggest that HEV host range and zoonotic spillover are unlikely to be determined by a single hepatocyte-specific receptor. Rather, cross-species infection may reflect the combined effects of viral entry determinants, virion properties, post-entry replication compatibility, host antiviral responses, and the broader cellular environment.

## 6 Host innate immune recognition of HEV

Immediately upon viral entry, HEV infection triggers a cascade of innate immune events. The activation of germline-encoded pattern-recognition receptors (PRRs) culminates in interferon production. However, as HEV is a non-cytopathic virus that can establish chronic infections, especially in immunocompromised patients, it has evolved to counter these innate immune responses (Kang and Myoung, 2017; Li et al., 2019; Lhomme et al., 2020).

Multiple PRR families have been identified as contributing to HEV recognition; however, cytoplasmic RIG-I-like receptors and Toll-like receptors are considered the principal sensors (Xu et al., 2021). The HEV genome possesses a 5′ m7G cap, but RIG-I and MDA5 are still able to detect HEV-derived RNA species, especially those produced during active replication (Devhare et al., 2013; Lhomme et al., 2020; Sooryanarain et al., 2020). Xu and colleagues demonstrated that activation of RIG-I suppresses HEV replication regardless of IFN production (Xu et al., 2017). The precise PAMP architecture within the HEV genome governs RIG-I activation. PAMPs derived from the 3′ UTR induce higher levels of IRF3 phosphorylation than those from the 5′ UTR. The U-rich region of the 3′ UTR has been identified as a potent RIG-I agonist. The poly(A) tail of the HEV genome further amplifies this response. However, Sooryanarain and colleagues identified these IFN responses as cell-type dependent. They reported a predominant type III IFN response in hepatocytes and a type I IFN response in enterocytes (Sooryanarain et al., 2020). Depletion of RIG-I and MDA5 has been shown to enhance HEV replication. This further confirms the functional relevance of RIG-I sensing in hepatic cells (Yin et al., 2017). Over the past decade, MDA5 has emerged as a potent inhibitor of HEV. Its overexpression induces transcription of ISGs and triggers STAT1 phosphorylation. This results in the activation of non-canonical IFN-like responses that inhibit HEV replication (Li Y. et al., 2020).

The HEV ORF1 putative papain-like cysteine protease domain has been demonstrated to downregulate MDA5-mediated IFN induction (Kim and Myoung, 2018). This designation presumes proteolytic processing of the ORF1 polyprotein, a premise that remains debated, as ORF1 is alternatively proposed to function as a single multifunctional ∼186 kDa polyprotein, with even its processing mechanism remaining contested (Pierce et al., 2023; Karpe, 2024).

TLRs also contribute to HEV sensing, especially in epithelial and immune cells. TLR2, TLR3, and TLR4 have been implicated in sensing HEV-derived molecular patterns (Devhare et al., 2013). Devhare and colleagues demonstrated that inflammatory cytokines (e.g., IL-6, IL-8, RANTES, and TNF-α) were robustly induced within 12 h of infection. This indicates that these early events are a response to viral entry and its structural components. In contrast, type I IFN gene expression was delayed and required active viral replication (Devhare et al., 2013). Soluble HEV capsid protein alone was not able to induce a cytokine response. This demonstrates that an intact capsid structure is required for TLR engagement (Devhare et al., 2013). HEV ORF3 protein has also been documented to counter TLR7-mediated signaling by disrupting the activation of NF-κB, JAK/STAT, and JNK/MAPK pathways (Lei et al., 2018).

Detection of HEV by PRRs is transduced into antiviral effector responses through signalosomes. These signalosomes are organized around the mitochondrial antiviral signaling protein (MAVS), the TBK1 and IKK kinases, and the nuclear transcription factors IRF3/IRF7 and NF-κB. Hepatitis A virus and hepatitis C virus engage proteases to cleave MAVS. However, HEV does not encode a functional protease capable of MAVS cleavage. Overall, MAVS protein localization and function remain unaltered during HEV infection (Yin et al., 2017; Mehnert et al., 2025). Yet, HEV actively dampens RLR-MAVS signaling. Accumulating evidence indicates that HEV not only downregulates RIG-I signaling but also co-opts MAVS and selectively limits inflammatory responses (Devhare et al., 2013).

Downstream of MAVS, TBK1 is decisive during HEV replication. Inhibition of TBK1 during HEV infection resulted in enhanced HEV replication, while overexpression of TBK1 significantly reduced viral infection (Xu et al., 2020). HEV ORF1 contains two putative domains that antagonize TBK1-dependent signaling (i.e., the X domain and PCP domain). The X domain inhibits RIG-I-induced phosphorylation, whereas the putative PCP domain deubiquitinates K63-linked ubiquitin from RIG-I and TBK1 (Nan et al., 2014b; Nan and Zhang, 2016). The ORF1 methyltransferase further suppresses RIG-I/MDA5-mediated IFN-β induction. It also blocks IRF3 and NF-κB p65 phosphorylation. This prevents the effective activation of the IFN-β promoter (Kang et al., 2018; Myoung et al., 2019; Xu et al., 2021). Overall, these findings indicate that HEV dampens but does not abolish IFN induction pathways (Feng and Lemon, 2014, 2019; Mehnert et al., 2025).

HEV employs multiple mechanisms to counter NF-κB signaling. The viral capsid protein blocks ubiquitin-mediated proteasomal degradation of IκBα, which in turn results in the inhibition of cellular NF-κB activity in ectopic expression systems (Surjit et al., 2012). ORF3, in ectopic expression systems, inhibits TRAF6 and NOD2 activity, resulting in reduced cytokine production (Lei et al., 2016). A recent study suggests that ORF2 interferes with NF-κB signaling by attenuating the induction of TNFAIP3 and IL6 (Mehnert et al., 2025).

The principal effector arm of HEV-triggered innate immunity is the IFN response. Robust ISG induction has been observed in HEV-infected cells and in chimpanzees. However, this induction is typically weaker than that observed in HCV infection, which is consistent with the lower levels of HEV viremia (Yu et al., 2010; Mehnert et al., 2025). Administration of exogenous type I IFNs does inhibit HEV replication, but to a lesser extent than that observed for HCV (Todt et al., 2016; Yin et al., 2017; Feng and Lemon, 2019). Recent research also indicates that HEV IFN resistance may operate predominantly at the level of upstream signaling antagonism, though direct modulation of STAT1 has also been reported (Feng and Lemon, 2019). The viral ORF3 protein inhibits IFN-α signaling by regulating STAT1 phosphorylation. This provides a direct molecular basis for IFN resistance (Dong et al., 2012; Lei et al., 2018). Functional studies have confirmed the antiviral relevance of type III IFN during HEV infection. Depletion of IFNLR1, but not IFNAR1, enhanced HEV replication. This indicates that type III IFN plays a more dominant role in controlling HEV in hepatocytes (Yin et al., 2017). HEV genotype 1 replication in decidual tissue explants was significantly inhibited by both IFN-λ1 and IFN-λ2. However, in placental tissues, only IFN-λ2 suppressed viral replication. This suggests tissue-specific responsiveness to type III IFN at the maternal-fetal interface (Gouilly et al., 2018; Feng and Lemon, 2019). Exogenous IFN-λ3 also exhibits moderate anti-HEV activity, though the effect is modest compared with IFN-α (Todt et al., 2016).

Several ISGs have been characterized for their role in restricting HEV replication. HEV infection induces robust ISG15 expression, although silencing of ISG15 enhances IFN-mediated antiviral signaling. This indicates that ISG15 acts as an immunomodulatory brake that reduces HEV sensitivity to type I IFN. Mechanistically, this brake operates at the level of type I IFN signaling rather than through direct modification of HEV components. ISG15 silencing potentiated type I IFN-mediated signaling and antiviral efficacy, with PKR and OAS1 identified as key effectors of ISG15-mediated IFN sensitivity, while ISG15 was dispensable for viral replication (Sooryanarain et al., 2017). Previously, ISG15-associated IFN resistance has been reported in chronic HCV infection (Chen et al., 2010). ISG15 induction may similarly serve a role during HEV persistence. Classical antiviral ISGs such as Mx proteins, OAS/RNase L, PKR, and ISG20 are also upregulated during HEV infection (Yu et al., 2010). IFIT1, another ISG, has been reported to bind HEV RNA and restrict HEV replication when overexpressed. However, HEV counters this by expressing an RNA-dependent RNA polymerase that sequesters IFIT1 to protect viral RNA (Pingale et al., 2019). Together, these ISG-level interactions highlight that HEV does not simply evade the interferon response upstream; it also actively counters individual effector molecules at the point of antiviral execution.

Interestingly, despite the persistent expression of ISGs induced by HEV, the virus continues to replicate. This indicates that a substantial fraction of induced ISGs is insufficient to eliminate the virus (Yin et al., 2017). Meyer and colleagues recently demonstrated that ISG expression is induced during HEV infection in both pig liver tissues and human HepaRG cells (Meyer et al., 2023).

Taken together, the HEV-host relationship emerges as a finely balanced interaction in which HEV triggers a sustained but ultimately insufficient IFN/ISG-mediated immune response that may permit persistent infection when antiviral control is inadequate.

## 7 Molecular mechanisms of HEV innate immune evasion

Although HEV does not encode a dedicated protease that cleaves MAVS, as observed for hepatitis A virus, it employs multiple mechanisms to modulate host innate immune signaling (Yin et al., 2017). Viral ORF1, ORF2, and ORF3 have each been implicated in immune modulation through distinct mechanisms, although the strength and direction of these effects can vary among experimental systems (Kang and Myoung, 2017; Kang et al., 2018; Feng and Lemon, 2019).

Since ORF1, ORF2, and ORF3 converge on overlapping nodes of a single signaling cascade, ie. spanning pathogen sensing, signalosome assembly, interferon induction, JAK/STAT signaling, and downstream effector execution, HEV evasion is described below protein by protein, with each subsection indicating the stage of the antiviral response most affected. Findings derived from ectopic expression of individual viral proteins are identified as such, since these systems may not recapitulate the stoichiometry and temporal regulation of viral proteins during authentic infection.

### 7.1 Viral antagonism of PRR signaling

The putative domains of ORF; Met, PCP, and X, are the principal effectors that blunt IFN induction. The MetYPCP region of ORF1 suppresses ISRE promoter activation and downstream ISG expression. It also inhibits STAT1 phosphorylation and nuclear translocation without affecting STAT2 phosphorylation. This indicates selective targeting of STAT1 within the JAK/STAT signaling pathway. MetYPCP of HEV-3 antagonizes STAT1 activation, whereas HEV-1 has no significant effect. This difference was mapped to the MetYPCP segment expressed independently of full-length polyprotein processing (Nan et al., 2014b; Bagdassarian et al., 2018), making primary sequence variation within the region, rather than differential ORF1 processing, the more plausible basis. (Figure 5).

**FIGURE 5:**
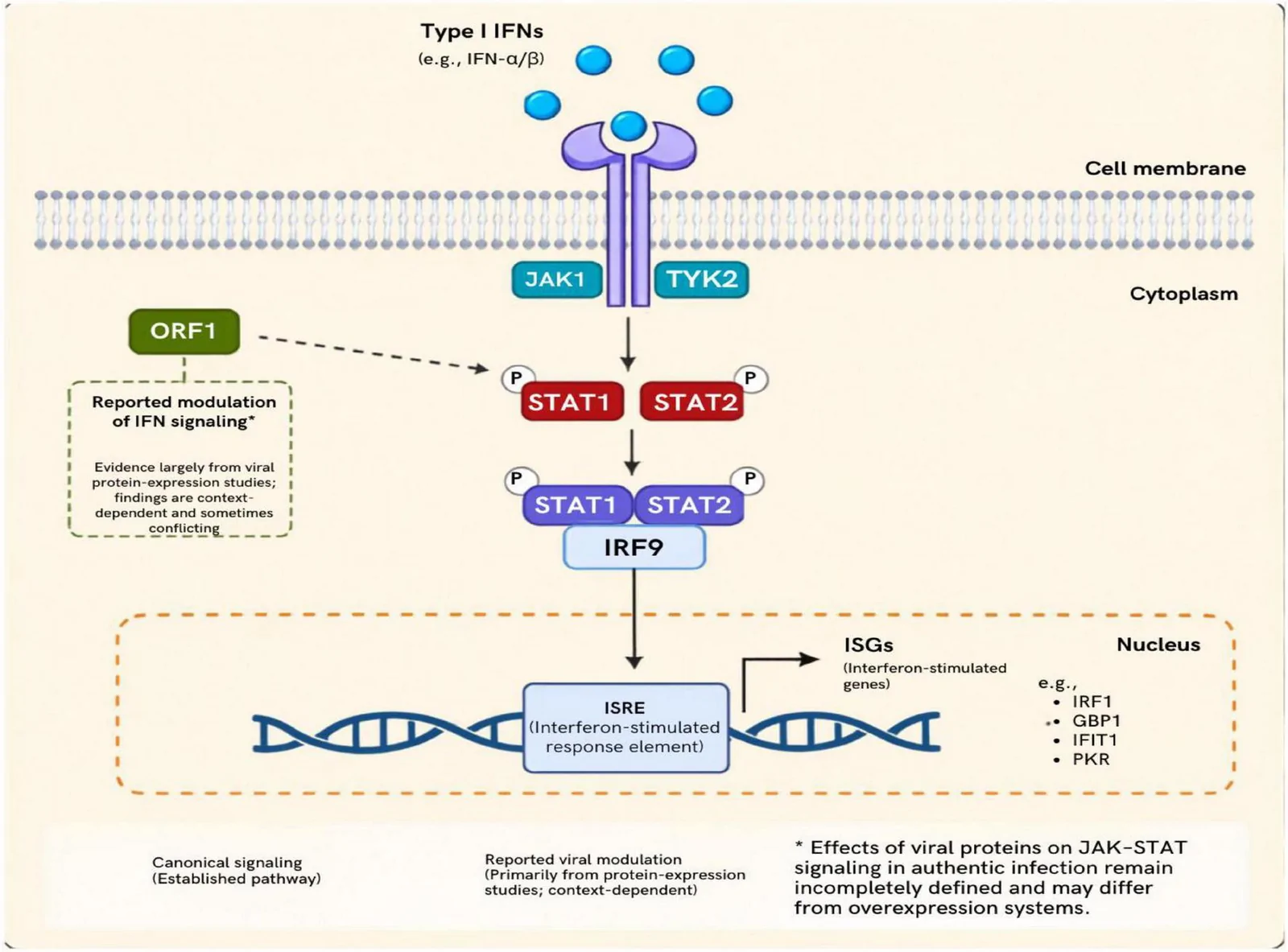
Type I interferon (IFN) signaling and reported modulation by HEV proteins. Type I IFN binding to its receptor activates JAK1 and TYK2, leading to phosphorylation of STAT1 and STAT2, formation of the STAT1–STAT2–IRF9 complex, and subsequent induction of interferon-stimulated genes (ISGs). Solid arrows represent the canonical JAK–STAT signaling pathway, whereas dashed arrows indicate reported modulation by HEV proteins. Reported effects of HEV proteins on IFN signaling are largely derived from viral protein expression studies and may be context-dependent; therefore, these interactions should not be interpreted as universally established mechanisms during authentic HEV infection. The extent and mechanisms of HEV-mediated modulation of JAK–STAT signaling during authentic infection remain incompletely defined.

The HEV Met domain significantly reduces RIG-I-induced IFN-β induction. It also negatively regulates NF-κB activity (Kang et al., 2018; Myoung et al., 2019; Xu et al., 2021). The X domain inhibits poly(I:C)-induced IRF3 phosphorylation. The X domain targets distinct molecular events, indicating independent intervention points within the RIG-I signalosome (Nan et al., 2014b). The putative PCP region also deubiquitinates K63-linked polyubiquitin chains from RIG-I and TBK1. This results in the inhibition of IFN induction. Studies indicate that the TBK1 activation state is a major determinant of HEV replication efficiency (Xu et al., 2020; Mehnert et al., 2025). The deubiquitinating activity of the putative PCP region also likely extends to other host proteins such as ISG15. This broadens the impact of this evasion strategy on the host immune landscape (Karpe and Lole, 2011; Feng and Lemon, 2014, 2019; Nan et al., 2014b; Nan and Zhang, 2016). Kim and Myoung demonstrated that the putative PCP region also contributes to the downregulation of MDA5 protein (Kim and Myoung, 2018).

The convergence of ORF1 antagonism on the MAVS–TBK1–IRF3 node is a key feature of HEV immune evasion. The parallel activities of X and the putative PCP region collectively regulate MAVS signaling without its cleavage, permitting RLR detection but an insufficient IFN response to clear the viral infection (Devhare et al., 2013; Yin et al., 2017; Feng and Lemon, 2019).

### 7.2 ORF2 as an immunomodulatory protein

ORF2 contributes to viral immune evasion at two levels. First, the secretion of a free glycosylated decoy isoform (ORF2^ S^) counters humoral immune responses. Moreover, the capsid surface of infectious virions is masked by a quasi-envelope acquired during egress (Feng and Lemon, 2014; Yin et al., 2018). The production of ORF2 in HEV-infected cells is far more complex than the translation of a single mRNA. Due to alternative translation initiation sites on the same subgenomic mRNA, two distinct ORF2 proteins are produced (Yin et al., 2018). Translation initiation at the first start codon produces a longer polypeptide with an N-terminal signal sequence, which directs ORF2^ S^ to the endoplasmic reticulum. ORF2^ S^ is then glycosylated and released via the secretory pathway. ORF2^ S^ is produced in significantly higher quantities than the capsid form (ORF2^ C^) (Yin et al., 2018; Walker, 2019). Translation of the actual capsid protein (ORF2^ C^) is initiated at a previously unrecognized internal start codon located 15 codons downstream. This yields a shorter protein that assembles into virions (Yin et al., 2018; Zhou et al., 2021). Although both isoforms of ORF2 overlap antigenically, they have distinct epitopes. ORF2^ S^ does not block HEV cell entry. However, ORF2^ S^ accumulates at very high concentrations in the serum during viral infection (Walker, 2019). This suggests a deliberately skewed translation strategy to divert immune resources. ORF2^ S^ acts as an antigen decoy that counters anti-capsid antibodies. The decoy mechanism is further corroborated by the structural analysis of broadly neutralizing human monoclonal antibodies. The study showed that these antibodies specifically recognize the non-glycosylated ORF2 in infectious particles but fail to bind the secreted ORF2^ S^. This indicates that ORF2^ S^ is antigenically distinct and supports its role as an immune sink (Ssebyatika et al., 2025).

In addition to its soluble decoy activity, ORF2 also contributes to intracellular immune evasion. ORF2 has been documented to inhibit cellular NF-κB activity by blocking proteasomal degradation of IκBα (Surjit et al., 2012). Mehnert and colleagues demonstrated that HEV ORF2 interferes specifically with NF-κB-mediated signal transduction downstream of MDA5. This results in reduced induction of TNFAIP3 and IL6 (Mehnert et al., 2025; Figure 6). Furthermore, the quasi-envelope acquired during viral egress also contributes to viral immune evasion (Feng and Lemon, 2014).

**FIGURE 6:**
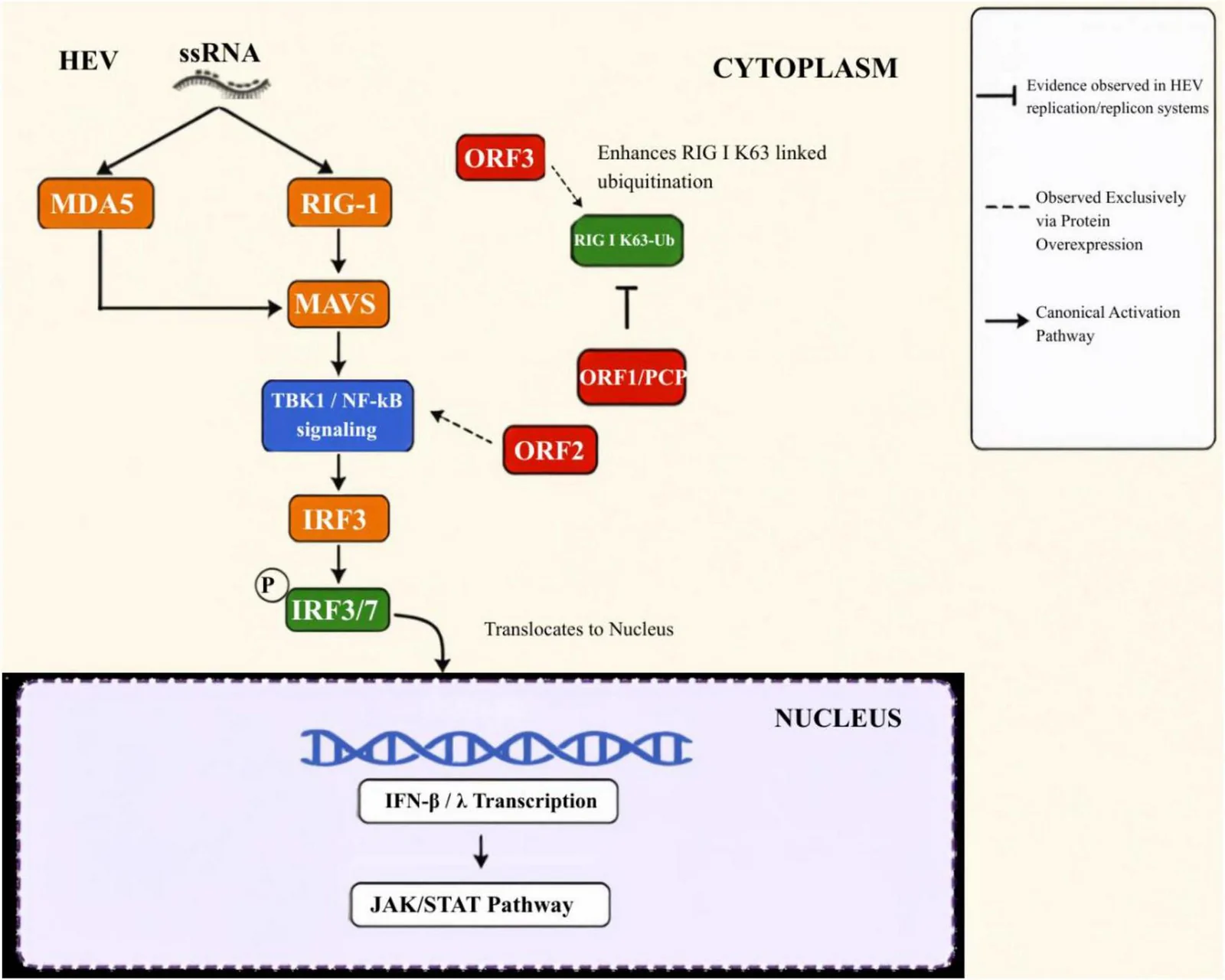
Reported effects of HEV proteins on type I interferon induction and signaling. The figure illustrates canonical RIG-I/MDA5–MAVS–TBK1/IRF3 signaling and subsequent IFN-β/λ production and JAK–STAT activation. Dashed arrows indicate effects reported primarily in viral protein-expression or overexpression studies, whereas T-bars indicate evidence observed in HEV replication/replicon systems.

### 7.3 ORF3 mediated immune evasion

Many mechanistic studies of individual HEV proteins have relied on ectopic protein expression, which may not fully recapitulate the stoichiometry and temporal regulation of viral proteins during authentic HEV infection, and the findings summarized below should be interpreted with this caveat in mind.

HEV ORF3 transiently activates NF-κB signaling during the early phase of infection. However, it inhibits this pathway during the late phase, creating a replication environment that shifts from pro-inflammatory to immunomodulatory as infection progresses (Xu et al., 2014; Lei et al., 2016; Glitscher and Hildt, 2021; Jiao et al., 2024). ORF3 reduces cytokine production by inhibiting TRAF6 and NOD2 activation and by decreasing the phosphorylation of IKKε and NF-κB (Lei et al., 2016). ORF3 has been reported to inhibit IFN-α signaling through its interaction with STAT1 in human alveolar cells (ectopic expression) (Dong et al., 2012; Lei et al., 2018; Xu et al., 2021). However, this effect has not been consistently observed across experimental systems. Mehnert and colleagues found that ORF2 exerted a stronger suppressive effect on antiviral signaling than ORF3 and did not observe substantial inhibition of IFN-induced JAK–STAT signaling by either protein in their experimental model (Mehnert et al., 2025). These findings highlight the context-dependent nature of ORF3-mediated immune modulation. ORF3 has also been reported to exert opposing effects on RIG-I-mediated antiviral signaling. While some studies have reported that ORF3 suppresses type I IFN signaling, Nan and colleagues found that ORF3 enhanced RIG-I ubiquitination and IFN induction (Nan et al., 2014a). These apparently divergent observations suggest that the effect of ORF3 on RIG-I signaling may depend on the experimental system, cellular context, or level and duration of ORF3 expression. Thus, the contribution of ORF3 to RIG-I signaling in the context of infection is not completely resolved (Lei et al., 2018).

ORF3 plays a critical role in the egress of quasi-enveloped HEV virions. The ORF3 protein interacts with the tumor susceptibility gene 101 protein through a classical PSAP late domain motif (Nagashima et al., 2011). It colocalizes with multivesicular bodies (MVBs) in the cytoplasm of infected cells, suggesting that HEV uses the MVB pathway for egress. Accumulating evidence indicates that HEV utilizes its multifunctional ORF3 protein to usurp host machinery for replication and egress. ORF3 acts as a late domain recruiter for the ESCRT system to facilitate quasi-enveloped viral release via multivesicular bodies. It also shares several structural features with class I viroporin that forms ion channels crucial for viral release (Ding et al., 2017). Overall, this temporary host membrane cloak shields the virus from neutralizing antibodies (Brüggemann et al., 2025; Fu et al., 2025).

ORF3 also modulates mitochondrial physiology in ways that may favor cell survival and viral persistence. However, the exact molecular mechanisms remain to be determined.

Current literature indicates that ORF3 protects against mitochondrial depolarization and cell death by upregulating VDAC and promoting hexokinase I–VDAC interaction, thereby preventing cytochrome c release (Moin et al., 2007; Glitscher and Hildt, 2021).

The interplay between viral egress and immune evasion remains an active area of investigation. Recent research, however, indicates that ORF3 viroporin activity likely stabilizes cellular homeostasis to permit prolonged particle production (Ding et al., 2017).

HEV infection may result in chronic hepatitis, especially in immunocompromised individuals. Patients with a history of solid organ transplants are particularly vulnerable to chronic HEV infection (Kamar et al., 2008). Clinical data and translational models confirm that immunosuppression-driven disruption of JAK–STAT and interferon signaling creates a permissive environment for viral persistence (Yin et al., 2017; Kinast et al., 2022; León-Janampa et al., 2023).

Collectively, the layered immune evasion strategies deployed by HEV spanning PRR antagonism, decoy antigen production, NF-κB suppression, and quasi-envelope shielding establish a molecular framework that enables the virus to persist within the host despite an active but ultimately insufficient immune response.

## 8 Conclusion

Hepatitis E virus does not rely on a single entry pathway or a single point of immune antagonism to establish infection; rather, it engages a distributed network of host receptors and cofactors and deploys innate immune evasion strategies across ORF1, ORF2, and ORF3 that together permit sustained viral replication despite an incomplete antiviral response. This layered and multifactorial architecture offers the most coherent current explanation for HEV’s capacity to establish both acute and persistent infection across a range of host and tissue contexts.

Several specific gaps limit the conclusions that can currently be drawn. First, the entry role of several candidate host factors remains genuinely unresolved rather than merely understudied. EGFR shows clear pleiotropic effects across the HEV life cycle: at the replication and release stages, EGFR exerts an antiviral effect, whereas HEV-mediated EGFR downregulation promotes viral replication and release, thereby favoring viral propagation highlighting the stage-dependent nature of EGFR function and the need to distinguish its candidate entry-cofactor activity from its effects on later replication and release (Woytinek et al., 2024), ATP5B is supported only by pull-down, mass spectrometry, and antibody/siRNA data against infectious virus, with its contribution to productive infection still undefined (Ahmed et al., 2016; Brüggemann et al., 2025). Additionally, the productive nature of extrahepatic infection is unresolved: HEV RNA, negative-strand intermediates, and antigen have been detected in renal, neurological, and gastrointestinal tissues, but whether this reflects genuine local replication and persistence, as opposed to passive dissemination of viral material from the circulation, has not been definitively established (Yadav and Kenney, 2023; Schlienkamp et al., 2026). ORF3’s effects on innate immune signaling are not internally consistent: it enhances RIG-I ubiquitination and downstream IFN induction in one system while suppressing TLR3/TLR7-dependent and STAT1-dependent signaling in others (Dong et al., 2012; Nan et al., 2014a; Lei et al., 2018; Corneillie et al., 2023). Given that most of this mechanistic work relies on ectopic protein expression rather than authentic infection, it remains unclear how much of this divergence reflects genuine pathway-specific or context-dependent biology versus the experimental system, the divergent effects of ORF3 on innate immune signaling may reflect differences in the experimental system versus the authentic infection context. How this divergence relates to the chronic infection seen in immunosuppressed hosts remains untested. Finally, the dual-module heuristic proposed in this review to organize the receptor evidence for hepatic tropism, while consistent with current data, has been assembled from findings generated in separate experimental systems and has not been tested as an integrated, falsifiable model in a single physiologically relevant system.

Addressing these gaps will require moving beyond ectopic expression and virus-like-particle systems toward authentic infectious HEV models; polarized or three-dimensional hepatocyte cultures and organoid models to directly test the dual-module heuristic and resolve EGFR’s role in entry versus replication; extrahepatic organoid or explant systems capable of distinguishing productive replication from passive viral carriage in kidney, neural, and placental tissue; and infectious-clone-based approaches to reconcile the conflicting reports of ORF3 function under conditions that better approximate natural infection kinetics and protein stoichiometry. Progress on these specific questions, more than incremental cataloging of additional candidate host factors, is likely to determine how quickly mechanistic insight into HEV entry, tropism, and innate immune evasion can be translated into targeted antivirals and improved diagnostics for a virus that continues to cause a substantial and likely underestimated global burden of disease. As a working hypothesis, the dual-module heuristic not only organizes the entry evidence but also frames unresolved questions regarding EGFR’s post-entry functions, NPC1’s mechanistic role, the structural basis of ORF1 polyprotein processing, and the nature of extrahepatic replication. Authentic-infection, organoid, and infectious-clone models are now positioned to address these questions.
